# In Situ Vaccines in the Era of Cancer Immunotherapy: Conceptual Innovation and Clinical Translation

**DOI:** 10.1002/advs.202509836

**Published:** 2025-08-19

**Authors:** Yiru Shi, Yuxuan Hou, Moustafa T. Mabrouk, Chengzhong Yu, Yannan Yang

**Affiliations:** ^1^ South Australian ImmunoGENomics Cancer Institute The University of Adelaide Adelaide SA 5005 Australia; ^2^ Australian Institute for Bioengineering and Nanotechnology The University of Queensland Brisbane QLD 4072 Australia

**Keywords:** cancer immunotherapy, clinical translation, immune response, immunogenic cell death, in situ vaccine, tumor microenvironment

## Abstract

Historically, directly injecting therapeutics into tumors has been deemed suboptimal and less favorable in clinical settings compared to systemic administration due to the inability to eradicate circulating/metastatic tumor cells until the emergence of the concept of in situ vaccine. In situ vaccine leverages patients’ own tumors as a pool of antigens to elicit systemic antitumor immunity (also known as “abscopal effect”) that aims to eliminate both primary and distal/metastatic tumors. One typical example of an approved product is Talimogene laherparepvec, an oncolytic virus approved in 2015 for treating advanced melanoma. To improve the effectiveness and the biosafety of in situ vaccines, various approaches have emerged, including new generations of oncolytic viruses, engineered bacteria, cytokine treatment, immune adjuvants, nanotechnology‐enabled formulations, and photo/radio‐therapies, showing remarkable promise in preclinical and clinical settings. These conceptual advances offer tailored solutions to challenges such as low antigen availability, limited immune activation, and side effects. In this review, we explore the current landscape of in situ cancer vaccines, categorizing them based on their functional formulations and highlighting their conceptual innovation in cancer immunotherapy. Additionally, a comprehensive discussion is provided of the existing clinical trials and our perspective on future clinical translation.

## Graphical abstract

In this review, the basic concepts of in situ vaccination are discussed, its advantages over conventional vaccines, and key historical milestones, from the first use of Coley's toxins to the most recent FDA‐approved in situ vaccine for bladder cancer. Major research advancements driving the clinical translation of this approach are also highlighted.

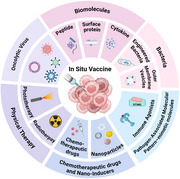

## 1 Introduction

In recent years, significant advancements have been made in the field of cancer immunotherapy.^[^ ^1^ ^]^ Many therapeutics have been developed for enhancing tumor immune response, such as monoclonal antibodies (e.g., immune checkpoint inhibitors),^[^ ^2^ ^]^ adoptive cell therapies (e.g., chimeric antigen receptor‐T cell therapy),^[^ ^3^ ^]^ and cancer vaccines (e.g., Sipuleucel‐T).^[^ ^4^ ^]^ These treatments can leverage the body's immune system to recognize and eliminate tumor cells.^[^ ^5^ ^]^ The systemic administration allows either an antigen‐specific or broad‐spectrum immune response, enhancing immune cell trafficking to tumors and facilitating tumor cell elimination at distant sites such as metastatic tumors.^[^ ^6^ ^]^ However, the therapeutic effect of these treatments by systemic administration is often limited by off‐tumor toxicity,^[^ ^6^ ^]^ immunosuppressive tumor microenvironment^[^ ^7^ ^]^ and insufficient drug penetration of blood vessel‐deficient tumors.^[^ ^8^ ^]^ While attempts have been made to engineer delivery system^[^ ^9^ ^]^ or microorganisms (e.g., bacteria) to enhance tumor specificity,^[^ ^10^ ^]^ and biocompatibility, suboptimal therapeutic activity is still inevitable.

In situ vaccines (ISV) are types of therapeutics that are delivered directly into the tumor site, transforming the patients’ own tumors as a tumor antigen source to elicit a broad‐spectrum of systemic antitumor immunity in a personalized manner.^[^ ^6^, ^11^ ^]^ A diverse range of agents, including bacteria,^[^ ^12^, ^13^ ^]^ virus,^[^ ^14^, ^15^ ^]^ small molecules,^[^ ^16^, ^17^ ^]^ live bacterial formulations,^[^ ^18^ ^]^ and immune‐stimulating nanoparticles^[^ ^19^, ^20^, ^21^ ^]^ are under investigation as potential tools for ISV. These agents contribute to the modulation of the tumor microenvironment (TME), enhancement of antigen presentation, or direct targeting of tumor cells to elicit a localized immune response.^[^ ^11^ ^]^ By harnessing the unique advantages of localized delivery, *ISVs* offer a promising path forward, with the potential to combine effective immune activation with reduced systemic toxicity.^[^ ^22^ ^]^

The concept of using localized treatments to stimulate systemic immune responses against cancer was first conducted by Dr. William Coley in the late 19th century.^[^ ^23^, ^24^ ^]^ He injected a mixture of dead bacteria, known as Coley's toxins, directly into tumors to induce an immune response that could attack the cancer. While the mechanism of action was not completely understood during that time, it was the first attempt to stimulate an anti‐tumor immune response.^[^ ^23^ ^]^ In 2015, the U.S. Food and Drug Administration (FDA) approved Talimogene laherparepvec (T‐VEC), an oncolytic virus (OVs), which was used for patients with advanced melanoma.^[^ ^25^ ^]^ T‐VEC is a genetically modified herpes simplex virus that only replicates within tumor cells and leads to their lysis.

**Figure 1:**
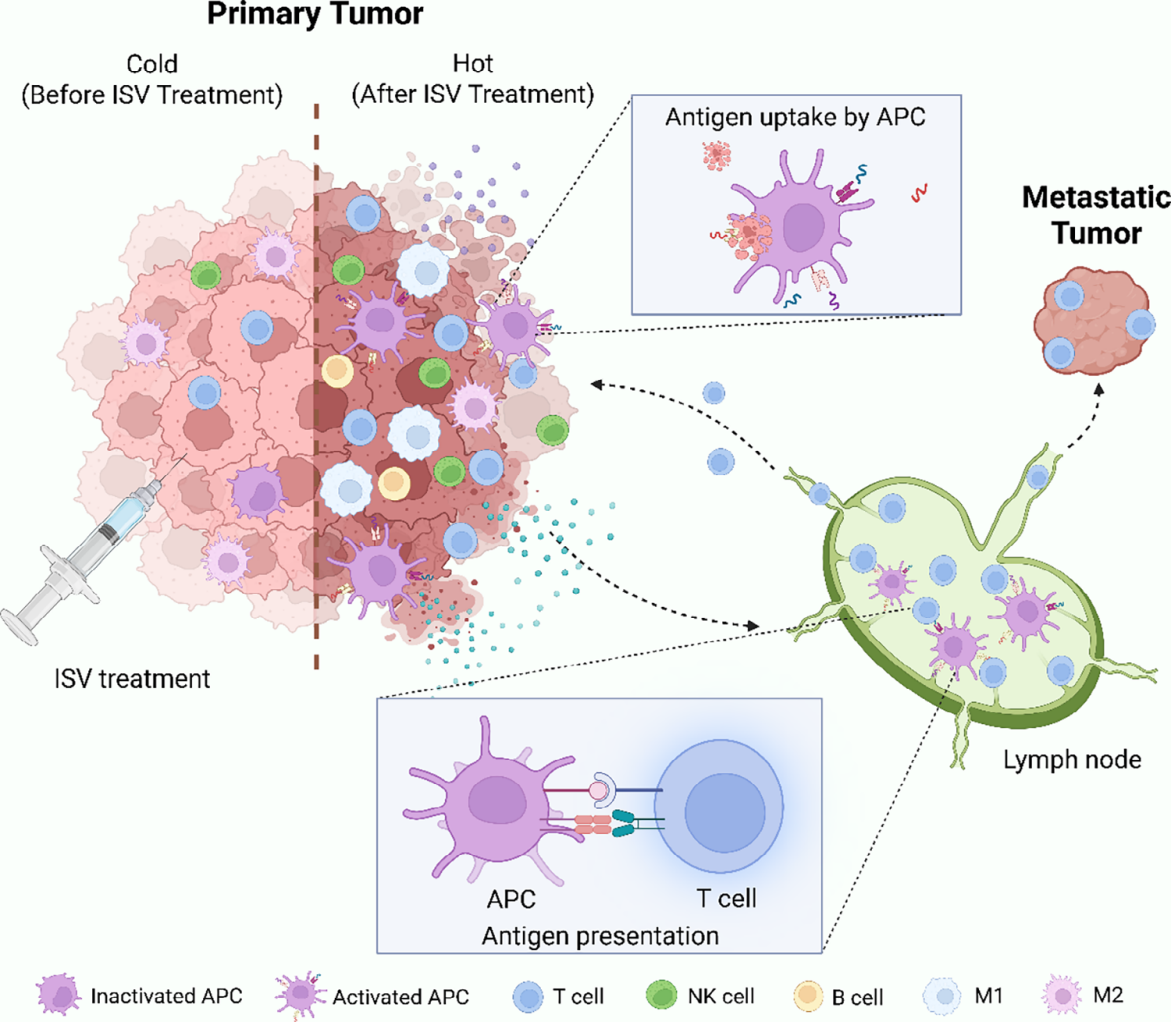
Schematic illustration of systemic antitumor immune responses induced by ISV treatment. **ISV**, In situ vaccine; **APCs,** antigen‐presenting cells; **M1**, M1‐like macrophages; **M2**, M2‐like macrophages. Created with BioRender.com.

Despite encouraging preclinical outcomes, the clinical translation of in situ vaccines remains limited by several challenges, including poor drug penetration into the tumor mass, suboptimal induction of immunogenicity, and the persistence of immunosuppressive mechanisms within the TME.^[^ ^11^, ^26^ ^]^ To address these barriers, a variety of innovative strategies have been proposed to enhance the efficacy and clinical applicability of in situ vaccine platforms. In this review, we summarize the latest advances aimed at overcoming these limitations and discuss the current progress toward clinical translation. Specifically, we describe various modulators developed for in situ vaccines based on their active components. These components play distinct roles in stimulating the immune system directly within the tumor microenvironment, thereby enhancing local immune activation and promoting systemic anti‐tumor immunity (**Figure** **1**). Bacteria and viruses can either directly activate immune responses or serve as biological vectors,^[^ ^24^, ^27^ ^]^ On the other hand, biomolecules and chemotherapeutics are utilized to modulate immune cells, induce tumor cell death, or reprogram cellular pathways.^[^ ^28^, ^29^ ^]^ Collectively, these diverse agents represent a broad approach for designing in situ vaccines with the potential for robust and targeted cancer immunotherapy.

Given the promise of this research field, a number of reviews have been published recently, focusing on delivery technology,^[^ ^30^ ^]^ biomaterial,^[^ ^22^ ^]^ nanotechnology^[^ ^31^ ^]^ and radiotherapy^[^ ^32^ ^]^ base in situ vaccine. Despite these insightful contributions, a thorough and systematic review of in situ vaccines has yet to be conducted. Our review addresses this gap by providing a systematic and detailed discussion of the current research innovation (**Table** **1**) and clinical trials, which will provide valuable insights to promote broad clinical application.

**Table 1:** Comprehensive Overview of In Situ Vaccines for Cancer Immunotherapy.

ISV Innovations	Functional agents/delivery system	Engineered Payload/Modification	Cancer Type	Immune cycle	Combination immunotherapy	Refs.
Oncolytic Virus	NDV	ICOSL	Melanoma	Tumor cell lysis enhances ICOS induced T‐cell activation	Anti‐CTLA‐4	[33]
	Adenovirus	Coated with tumor peptide (MHC‐I epitopes)	Melanoma	Tumor cell lysis, improved tumor‐specific targeting		[34]
	Adenovirus	Coated with tumor peptide (MHC‐I and MHC‐II epitopes)	Melanoma	Tumor cell lysis, enhanced activation of CD4⁺ and CD8⁺ T cells		[35]
	Adenovirus	Encode CD40L, OX40L, coated with tumor peptide (MHC‐I epitopes)	Melanoma	Tumor cell lysis; enhanced activation of APCs, promoting T‐cell proliferation and survival.	Anti‐PD‐1	[36]
	Adenovirus	Encode SIRPα‐Fc, Siglec10‐Fc, TIGIT‐Fc	Colon cancer; Breast cancer	Tumor cell lysis		[37]
	Adenovirus	Cancer cell membrane coating	Melanoma; Lung cancer	Tumor cell lysis		[38]
	Adenovirus	None (antigen depot)	Glioblastoma	Tumor cell lysis		[39]
	Vaccinia Virus	GM‐CSF, IL‐21	Pancreatic cancer; Colon cancer	Tumor cell lysis	Anti‐PD‐1	[40]
	Vaccinia Virus	IL‐12	Lung cancer	Tumor cell lysis	Anti‐PD‐1	[41]
Cytokine	IL‐23 + IL‐36γ + OX40L	IL‐23 + IL‐36γ + OX40L mRNA	Colon cancer; Liver cancer; Melanoma; Lymphoma	Tumor cell lysis	Anti‐PD‐L1; Anti‐PD‐1; Anti‐CTLA‐4	[42]
	IL‐2	Collagen‐anchored IL‐2 fusion protein	Breast cancer; Colon cancer; Melanoma	Tumor cell lysis	Anti‐PD‐1; CAR‐T cell therapy; TA99	[43]
	IL‐12	Phosphoserine‐tagged IL‐12 fused with alum	Breast cancer; Colon cancer; Melanoma; Fibrosarcoma	Tumor cell lysis	Anti ‐PD‐1	[44]
Surface Protein	anti‐HER2, SLAMF7		Breast cancer	Improve phagocytes recognition and phagocytosis, sensitize tumor cells to CD47 blockade	Anti‐PD‐1; Anti‐CD47	[45]
	anti‐OX40 antibody		Breast cancer; Colon cancer, Melanoma, Lymphoma	Promote Teff activation and inhibit Treg function.	Anti‐CTLA‐4	[46]
Peptide	LTX‐315		Sarcoma	Tumor cell lysis, ICD		[47]
	α‐melittin‐NP		Melanoma	Tumor cell lysis, ICD		[48]
	DNPs		Melanoma	Tumor cell lysis, ICD		[49]
Engineered Bacteria	E. coli Nissle 1917	CD47 nanobody	Lymphoma; Breast cancer; Melanoma	Enhance macrophage phagocytosis		[12]
	E. coli Nissle 1917	CXCL16 and CCL20 chemokines	Lymphoma; Colorectal cancer; Breast cancer	Enhance recruitment of CD8^+^ T cells and DC		[50]
	E. coli Nissle 1917	Neoantigen, LLO	Colon cancer; Melanoma	Promote APC cross‐presentation and neoantigen‐specific CD4^+^/CD8^+^ T cell responses		[51]
	E. coli Nissle 1917	Cyclic di‐AMP	Melanoma; B‐cell lymphoma	Activate APCs		[52]
	E. coli Nissle 1917	L‐arginine	CMolon cancer; melanoma	Enhance T cell activation and proliferation	Anti‐PD‐L1	[53]
OMV	Salmonella Typhimurium–derived OMVs		Melanoma	Activate APCs, remodel TME		[54]
	Photosynthetic bacteria–derived OMVs		Breast cancer, Melanoma	Activate APCs, remodel TME		[55]
	Fusobacterium nucleatum–derived OMVs		Breast cancer; Oral squamous cell carcinoma	Activate APCs, remodel TME	Anti‐PD‐L1	[56]
Stimulator of Interferon genes (STING) Agonist	DMXAA		Mesothelioma	Activate APCs,		[57]
	ADU‐S100		Advanced/metastatic solid tumors or lymphomas	Activate APCs	DNA methyltransferase inhibitor (5AZADC)	[58]
	2′3′‐cGAMP		Breast cancer; Melanoma	Activate APCs	Anti‐PD‐1; Anti‐PD‐L1	[59]
	cyclic di‐AMP		Breast cancer	Activate APCs		[60]
	Mn^2^⁺		Colorectal cancer; Melanoma	Activate APCs	Anti‐PD‐1	[61]
TLR Agonist	Poly(I:C)		Lung cancer model	TLR3 agonist modulate TAMs toward M1‐like macrophages		[62]
	Glucopyranosyl lipid A		Melanoma; Glioblastoma	Activate APCs		[63]
	R848		Breast cancer, Colorectal cancer	TLR7/8 agonist activates APCs	adoptive cell therapy, lentiviral vector	[64]
	CpG		Breast cancer;Colon cancer; Melanoma	TLR9 agonists activate APCs	anti‐PD‐1	[65]
Chemotherapeutic drug	DOX		colorectal cancer	Tumor cell death, ICD	DPPA‐1	[66]
	Combretastatin A‐4‐phosphate		Hepatocellular carcinoma	Dystroy tumor vasculature	Anti‐PD‐L1	[67]
Nano‐Inducers	CaH_2_ nanoparticles		Breast cancer; Colorectal cancer	Tumor cell death, ICD	Anti‐CTLA4	[20]
	F@D‐CHTP SN‐MF		Breast cancer	Tumor cell death, ferroptosis, cuproptosis, ICD	STING agonist and VEGFR inhibitor	[68]
	NaCl nanoparticles		Prostate cancer; Glioblastoma; Melanoma, Bladder carcinoma; Squamous cell carcinoma	Tumor cell death, pyroptosis ICD		[69]
	CaCO_3_ nanoparticles		Breast cancer	Tumor cell death, pyroptosis, ICD	Anti‐PD‐L1	[70]
Phototherapy	PTT Sensitizer‐IR820		Breast cancer; Melanoma	Tumor cell death, ICD	Anti‐PD‐L1	[71]
	AIEgen‐coupled upconversion nanoparticles (AUNPs)		Melanoma	Tumor cell death, ICD	Anti‐PD‐1	[72]
	DNPs@CM		Cervical cancer	Tumor cell death, ICD	Alum	[73]
Radiotherapy	Heterogeneous RT dose		Melanoma; Prostate cancer	Tumor cell death	Anti‐CTLA‐4, anti‐PD‐L1	[74]
	Hf_12_‐DBA as radio enhancers		Colorectal cancer	Tumor cell death, ICD	Anti‐PD‐L1	[75]

## 2 Conceptual Innovations

### 2.1 Oncolytic Virus

Oncolytic viruses (OVs) represent a dynamic class of immunotherapeutic agents that selectively infect and lyse tumor cells, orchestrating robust antitumor immunity.^[^ ^76^ ^]^ By replicating within malignant cells, OVs induce immunogenic cell death (ICD), liberating tumor‐associated antigens and damage‐associated molecular patterns (DAMPs).^[^ ^27^ ^]^ These molecules activate antigen‐presenting cells (APCs), particularly dendritic cells (DCs), fostering CD8⁺ T‐cell priming and infiltration.^[^ ^27^, ^77^ ^]^ Concurrently, OVs engage innate immunity by stimulating pattern recognition receptors (PRRs), such as toll‐like receptors (TLRs), on immune cells, eliciting proinflammatory cytokines (e.g., IL‐6, TNF‐α) and chemokines.^[^ ^78^ ^]^ These mediators recruit natural killer (NK) cells, M1‐polarized macrophages, and neutrophils, transforming the tumor TME into a hub for anti‐tumor immune response. Despite their transformative potential, challenges such as off‐target replication in healthy cells,^[^ ^79^ ^]^ immune neutralization, and limited efficacy in immunosuppressive TMEs constrain clinical translation.^[^ ^15^ ^]^

The FDA approval of Talimogene laherparepvec (T‐VEC), an engineered herpes simplex virus type 1 (HSV‐1) expressing granulocyte‐macrophage colony‐stimulating factor (GM‐CSF), marked a pivotal milestone in oncolytic virotherapy.^[^ ^80^ ^]^ As the first oncolytic OV approved for melanoma, T‐VEC induces immunogenic cell death (ICD) and stimulates localized immune responses within the TME. Despite its success, T‐VEC's efficacy is limited in immunologically “cold” tumors or immunocompromised hosts, where immunosuppressive barriers hinder robust immune activation.

To overcome these challenges, next‐generation OVs are engineered to deliver immunomodulatory payloads or synergize with immune checkpoint inhibitors, reprogramming the TME to promote systemic anti‐tumor immunity.^[^ ^81^ ^]^ In the case of Newcastle disease virus (NDV), its therapeutic effect is reduced in larger tumors, necessitating additional immune pathway targeting to bolster efficacy. Given the upregulated inducible T‐cell co‐stimulator (ICOS) after NDV treatment, recombinant NDV expressing ICOS ligand (ICOSL) was designed for targeting tumor cells.^[^ ^33^ ^]^ This strategy promotes regression of both injected and abscopal lesions, with enhanced tumor rejection when combined with CTLA‐4 blockade. By integrating targeted immunomodulation, these advanced OVs offer a pathway to overcome immunosuppressive barriers and achieve durable anti‐tumor responses.

To enhance the specificity of antitumor immune responses, researchers have developed Peptide‐coated Conditionally Replicating Adenovirus (PeptiCRAd), an oncolytic adenovirus platform coated with tumor‐specific peptides, such as MHC‐I epitopes or patient‐derived tumor epitopes.^[^ ^34^ ^]^ This platform efficiently stimulates tumor‐specific immune responses by presenting these peptides to immune cells. In preclinical studies, PeptiCRAd coated with MHC‐I epitopes demonstrated significant efficacy against B16‐F10 melanoma in mouse models, resulting in substantial accumulation of CD8^+^ T cells in the spleen and lymph nodes, indicating robust immune activation.

**Figure 2:**
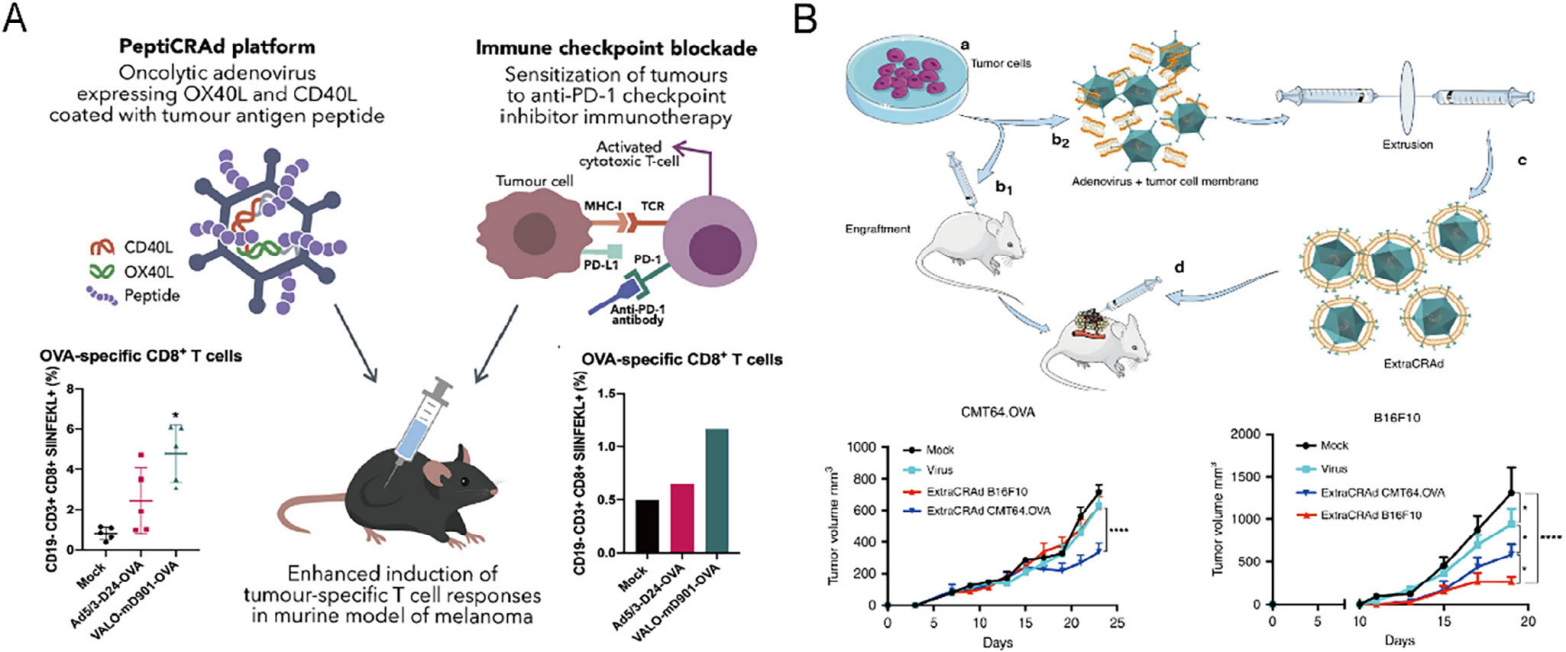
A) Schematic illustration of PeptiCRAd design and mechanism. An oncolytic adenovirus is coated with tumor‐specific peptides and genetically engineered to express CD40L and OX40L. CD40L activates APCs to enhance tumor antigen presentation, while OX40L promotes proliferation and survival of CD8⁺ T cells. Reproduced with permission.^[^ ^36^ ^]^ Copyright 2021, Elsevier. B) Schematic illustration of ExtraCRAd. Conditionally replicating adenoviruses coated with cancer cell membranes act as a diverse antigen source, presenting tumor‐associated antigens to immune cells. This approach elicits tumor‐specific immune responses in melanoma and lung cancer models. Reproduced with permission.^[^ ^38^ ^]^ Copyright 2019, Springer Nature.

Building on this versatile cancer vaccine platform, several advancements have optimized its immunogenicity. One approach incorporates both MHC‐I and MHC‐II restricted tumor epitopes to broaden the immune response, engaging both CD8^+^ and CD4^+^ T cells for a more comprehensive antitumor effect. Additionally, incorporating pathogen‐specific epitopes, such as toxoid peptides, sustains CD8^+^ T cell‐mediated responses by activating memory CD4^+^ T cells, thereby enhancing long‐term immunity.^[^ ^35^ ^]^ Another significant improvement involves encoding the adenovirus with immunostimulatory molecules, specifically CD40 ligand (CD40L) and OX40 ligand (OX40L), to amplify CD8^+^ T cell activation and expansion. This modified virus activates both innate and adaptive immune responses by licensing antigen‐presenting cells (APCs) via CD40L and promoting T cell proliferation and survival via OX40L (**Figure** **2**A).^[^ ^36^ ^]^

To further improve tumor targeting, researchers developed Extra Conditionally Replicating Adenovirus (ExtraCRAd), where the adenovirus is coated with cancer cell membranes that serve as a diverse antigen pool (Figure 2B).^[^ ^38^ ^]^ This led to a more specific antitumoral response after virus infection in the melanoma and lung cancer models. However, the limited and unsustainable antigen supply can hinder long‐term therapeutic efficacy. To address this, an oncolytic adenovirus reservoir (OAR) was developed as a novel strategy for glioblastoma treatment.^[^ ^39^ ^]^ The OAR is created by freezing oncolytic adenovirus‐loaded tumor cells in liquid nitrogen, enabling sustained viral release and continuous tumor cell lysis, thereby maintaining prolonged immune stimulation.

Apart from targeting tumor cells, some studies indicated that enriched immune cells, such as macrophages and T cells, within TME could be a target for cancer therapy. For example, recombinant oncolytic adenoviruses (OAds) were generated to deliver OAd‐SIRPα‐Fc, OAd‐Siglec10‐Fc, and OAd‐TIGIT‐Fc proteins, which can bind to CD47, CD24, or CD155, respectively.^[^ ^37^ ^]^ The three different recombinant OAd were assessed in three tumor model:CT26 colony tumor model, the MC38 colony tumor model, and 4T1 breast cancer tumor model. OAd‐SIRPα‐Fc and OAd‐Siglec10‐Fc could achieve enhanced macrophage‐dominated antitumor efficacy, whereas TIGIT‐Fc could enhance CD8^+^ T‐cell‐mediated immune response.

Another explored virus is the Vaccinia virus (VV), which exemplifies a versatile oncolytic platform due to its lack of requirement for a specific surface receptor.^[^ ^82^ ^]^ Studies have been attempted to enhance its antitumor efficacy and safety.^[^ ^83^ ^]^ For instance, deletion of VV N1L protein (VVΔTKΔN1L) has been demonstrated to reduce tumor metastatic and enhance survival through enhancing circulating NK cells and upregulating inflammatory cytokines in the lung cancer model.^[^ ^84^ ^]^ Arming VVΔTKΔN1L with IL‐12 has shown efficacy in pancreatic and head and neck cancer models. In their following study, by modifying the virus B5R protein, the ability of VV to spread within and between tumors is improved. This VV vector, named VVLDTK‐STCDN1L‐IL12 can sensitize lung cancer to a‐PD1 therapy.^[^ ^41^ ^]^ Furthermore, deletion of the A49 protein of VV and arming with GM‐CSF and interleukin‐21 (IL‐21), could enhance tumor selectivity and both innate and adaptive immunity in pancreatic and colon cancer models.^[^ ^40^ ^]^

### 2.2 Biomolecules

Biomolecules, including cytokines, surface proteins, and peptides, have emerged as a cornerstone of cancer immunotherapy due to their inherent immunogenicity and versatility in modulating immune responses within the tumor microenvironment. These biomolecule‐based ISVs leverage the body's immune system to target and eliminate malignant cells, offering a promising alternative to conventional therapies like chemotherapy and radiation, which often lack specificity and cause significant side effects.^[^ ^85^ ^]^ By directly engaging immune cells at the tumor site, biomolecules enhance immune recognition, promote antitumor effects, and overcome immunosuppressive barriers commonly found in solid tumors.^[^ ^86^ ^]^ Their ability to stimulate both innate and adaptive immunity positions them as critical tools in developing personalized and effective cancer treatments, with ongoing research focused on optimizing delivery and efficacy.^[^ ^87^ ^]^

#### 2.2.1 Cytokine

Cytokines, such as interleukin‐2 (IL‐2), interleukin‐12 (IL‐12), and interferons (IFNs), are proinflammatory molecules extensively studied in immunotherapy for their capacity to generate adaptive immune responses and alleviate immunosuppression.^[^ ^88^, ^89^ ^]^ In a clinical trial for triple‐negative breast cancer (TNBC), PD‐L1 blockade therapy showed limited efficacy due to the poor immunogenicity of TNBC tumors.^[^ ^90^ ^]^ Systemic administration of cytokines, while effective, is often hindered by toxicity to healthy tissues.^[^ ^89^, ^91^ ^]^ Consequently, localized delivery of cytokines within the TME has emerged as a promising strategy to maximize drug exposure at the tumor site while minimizing systemic toxicity.^[^ ^44^ ^]^ However, direct injection of cytokine proteins into tumors has demonstrated limited tumor inhibition due to rapid clearance, which reduces their ability to sustain prolonged immune stimulation within the TME.^[^ ^92^ ^]^ To address these challenges, researchers have developed methods such as encapsulating cytokines in lipid nanoparticles, delivering mRNA or plasmids encoding cytokines, and modifying cytokine proteins to enhance retention and efficacy.^[^ ^93^ ^]^

One approach involves delivering cytokine mRNA to reduce toxicity associated with direct cytokine administration. For example, mRNAs encoding interleukin‐23 (IL‐23), interleukin‐36 gamma (IL‐36γ), and OX40 ligand (OX40L) were encapsulated in lipid nanoparticles (LNPs).^[^ ^42^ ^]^ In this delivery system, IL‐23 and IL‐36γ can initiate immune responses by inducing inflammation within the TME, while OX40L, a T‐cell costimulatory molecule, enhances T‐cell activation and proliferation. This localized delivery maximizes therapeutic effects at the tumor site while minimizing systemic toxicity. By transforming the immunosuppressive TME into an inflamed state, the tumor becomes more susceptible to immune attack, particularly when combined with checkpoint inhibitors like anti‐PD‐L1. However, achieving sufficient cytokine accumulation and sustained release within the TME remains a challenge, even with mRNA or plasmid‐based approaches.

**Figure 3:**
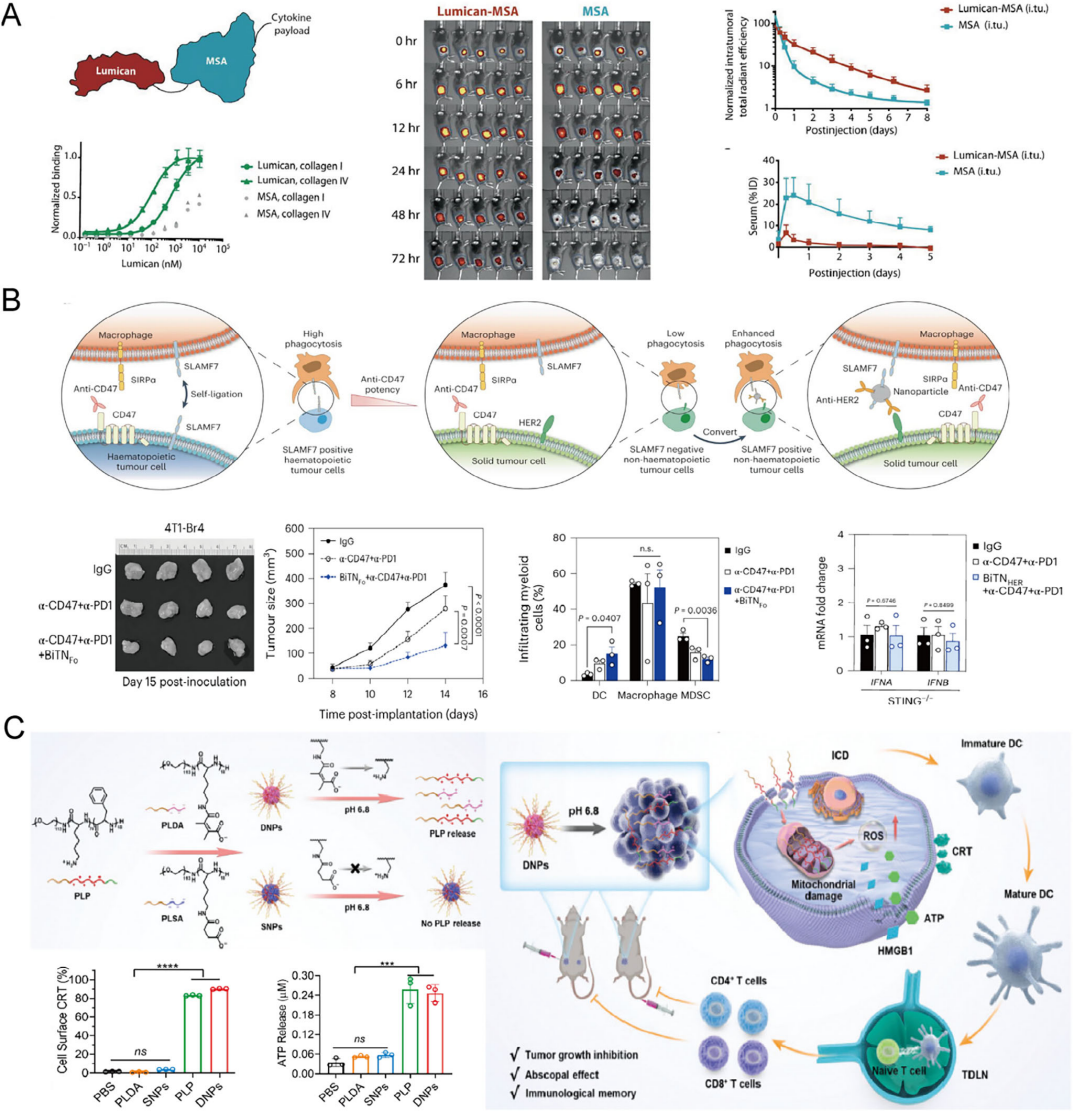
A) Schematic illustration of the Lumican‐MSA conjugate highlighting its binding affinity to collagen types I and IV for targeted cytokine delivery. Time‐lapse microscopy images demonstrate the progressive tumor accumulation of Lumican‐MSA over 72 h. Lumican‐fused cytokines IL‐2 and IL‐12 exhibit sustained activity at the tumor site and reduced systemic exposure compared to unlinked MSA. Reproduced with permission.^[^ ^43^ ^]^ Copyright 2019, American Association for the Advancement of Science. B) Schematic of the bispecific nanobioconjugate (BiTNHER) designed to target SLAMF7 and HER2, enhancing macrophage‐mediated phagocytosis of tumor cells. Digital imaging and tumor growth curves illustrate significant tumor inhibition following treatment with Bother combined with anti‐CD47 and anti‐PD‐L1 therapies. Enhanced expression of type I interferons in intratumoral F4/80⁺ macrophages after triple‐combination treatment indicates activation of the STING pathway. Reproduced with permission.^[^ ^45^ ^]^ Copyright 2022, Springer Nature. C) Chemical structure and schematic illustration of the pH‐responsive mechanism governing the release of PLP from DNPs in the mildly acidic tumor microenvironment. Released PLP triggers immunogenic cell death, promotes dendritic cell maturation, and robust T‐cell‐mediated antitumor immunity. Reproduced with permission.^[^ ^49^ ^]^ Copyright 2025, Elsevier.

To improve cytokine retention, researchers have leveraged the high collagen expression in tumors.^[^ ^94^ ^]^ Lumican, a collagen‐binding protein, was fused with IL‐2 and IL‐12 to enhance cytokine retention at the tumor site.^[^ ^43^ ^]^ Compared to unlinked mouse serum albumin, Lumican‐fused cytokines exhibited increased tumor accumulation and reduced serum levels (**Figure** **3**A). However, treatment with Lumican‐fused IL‐2 and IL‐12 only delayed tumor growth in mice. Combining this approach with immunotherapies such as anti‐PD‐L1, chimeric antigen receptor T‐cells (CAR‐T), and TA99 (anti‐TRP1) significantly improved survival rates across various tumor models, indicating an enhanced T‐cell response mediated by IL‐2 and IL‐12. This strategy holds potential for delivering immunomodulatory agents with prolonged retention at tumor sites, but the limited collagen in the TME restricts the effective dose. In a subsequent study, cytokines fused with alum and Fam20C (a single kinase) were developed.^[^ ^44^ ^]^ These alum‐tethered cytokines remain at the tumor site, providing sustained, controlled release and eliciting a more robust immune response with a single dose compared to previous methods.

#### 2.2.2 Surface Protein

Surface proteins are engineered to enhance innate and adaptive immune responses by improving immune recognition and tumor cell destruction.^[^ ^95^ ^]^ For instance, delivering Annexin V blocks phosphatidylserine (PS)‐mediated phagocytosis of apoptotic cells, preventing immune evasion.^[^ ^96^ ^]^ Another approach utilizes signaling lymphocytic activation molecule family member 7 (SLAMF7) to augment macrophage phagocytosis mediated by CD47 blockade (Figure 3B).^[^ ^45^ ^]^ A bispecific nanobioconjugate platform targeting SLAMF7 and human epidermal growth factor receptor 2 (HER2), named BiTNHER, significantly increased lymphocyte infiltration in the HER2‐overexpressing TUBO tumor model when combined with anti‐CD47 therapy. Incorporating anti‐PD‐L1 into a triple combination therapy activated the STING pathway in tumor‐associated macrophages (TAMs), resulting in elevated IFN‐α and IFN‐β mRNA expression. This platform's flexibility allows adaptation to other tumor models, such as triple‐negative breast cancer, by substituting HER2 with folate, which is overexpressed in 4T1 cells, a TNBC model.

Antibodies targeting surface receptors also demonstrate effective antitumor immune responses. OX40 (CD134), a costimulatory receptor in the tumor necrosis factor receptor (TNFR) superfamily, is predominantly expressed on activated effector T cells and regulatory T cells (Tregs). Upon binding its ligand or agonistic antibodies, OX40 signaling promotes Teff proliferation, survival, and memory formation while suppressing Treg immunosuppressive functions, enhancing antitumor immunity. Sagiv‐Barfi et al. developed an effective ISV strategy by combining intratumoral administration of CpG oligodeoxynucleotide, a TLR9 agonist, with an agonistic anti‐OX40 antibody.^[^ ^46^ ^]^ This approach induces OX40 expression on intratumoral CD4^+^ T cells, sensitizing them for further stimulation. The combination therapy triggered robust systemic antitumor immune responses, leading to regression of both injected and distant untreated tumors across various cancer types, including lymphoma, breast carcinoma, colon cancer, and melanoma. It also reduced tumor burden and metastases while improving survival in a spontaneous breast cancer model. Notably, low‐dose local administration minimized systemic toxicity, highlighting its potential for clinical translation.

#### 2.2.3 Peptide

Cationic amphipathic peptides are engineered to exploit the negatively charged surfaces of cancer cells, enabling targeted binding and membrane permeabilization.^[^ ^97^ ^]^ These oncolytic peptides induce tumor cell lysis and immunogenic cell death.^[^ ^48^, ^98^ ^]^ The release of damage‐DAMPs recruits dendritic cells and primes tumor‐specific cytotoxic T lymphocytes, positioning oncolytic peptides as potent candidates for in situ vaccines. LTX‐315, a synthetic 9‐mer peptide derived from bovine lactoferricin, is optimized for oncolytic activity and has advanced to phase II clinical trials.^[^ ^47^ ^]^

Melittin, a peptide from bee venom, induces apoptosis and ICD by disrupting tumor cell membranes.^[^ ^21^, ^48^ ^]^ However, its clinical use is limited by haemolytic toxicity and a narrow therapeutic window.^[^ ^99^ ^]^ Encapsulation in nanoparticles or polymer carriers has reduced systemic toxicity while preserving antitumor efficacy.^[^ ^21^, ^48^, ^100^ ^]^ For example, α‐melittin nanoparticles (α‐melittin‐NPs) target lymph nodes, promoting systemic immune responses by releasing tumor antigens and activating antigen‐presenting cells, leading to enhanced antigen‐specific CD8^+^ T‐cell responses.^[^ ^48^ ^]^

In another approach, a cationic oncolytic polypeptide (PLP) is complexed with a pH‐sensitive anionic polypeptide (PLDA) to form charge‐shielded nanoparticles (DNPs) stable at physiological pH.^[^ ^49^ ^]^ In the mildly acidic TME (pH 6.5–7.0), acid‐labile bonds within the nanoparticle matrix cleave, releasing PLP in situ. The released PLP induces tumor cell destruction through membrane lysis, mitochondrial disruption, and increased reactive oxygen species (ROS), triggering robust ICD (Figure 3C). These environmentally responsive nanoparticles highlight the potential of peptides as precise, localized ISVs.

### 2.3 Bacteria

Since the late 19th century, when Dr. William Coley used inactivated bacteria to stimulate immune responses in cancer patients,^[^ ^23^, ^24^ ^]^ bacterial‐based therapies have been recognized as a viable approach for cancer immunotherapy.^[^ ^24^ ^]^ Bacteria trigger robust immune responses by engaging both innate and adaptive immunity through pathogen‐associated molecular patterns (PAMPs), such as lipopolysaccharides (LPS), peptidoglycan, and flagellin.^[^ ^10^ ^]^ These PAMPs activate immune cells, including macrophages, dendritic cells (DCs), and T cells, transforming the immunosuppressive tumor microenvironment (TME) into an immune‐active state conducive to antitumor activity.^[^ ^101^, ^102^ ^]^

The hypoxic nature of tumors makes them ideal for colonization by anaerobic bacteria.^[^ ^103^ ^]^ For example, genetically modified *Salmonella Typhimurium* (VNP20009) preferentially proliferates in tumors, achieving a tumor‐to‐normal organ bacterial ratio of 10000:1 following intravenous injection.^[^ ^104^ ^]^ The immunosuppressive TME further protects bacteria from immune clearance, allowing them to exert therapeutic effects.^[^ ^105^ ^]^ Beyond direct immune activation, bacteria can be engineered to produce or deliver therapeutic molecules, such as immune checkpoint inhibitors,^[^ ^106^ ^]^ cytokines,^[^ ^107^ ^]^ or other antitumor agents,^[^ ^106^ ^]^ serving as efficient delivery platforms. By harnessing their innate immunogenicity, bacteria offer a unique and promising strategy for cancer therapy.

#### 2.3.1 Engineered Bacteria

**Figure 4:**
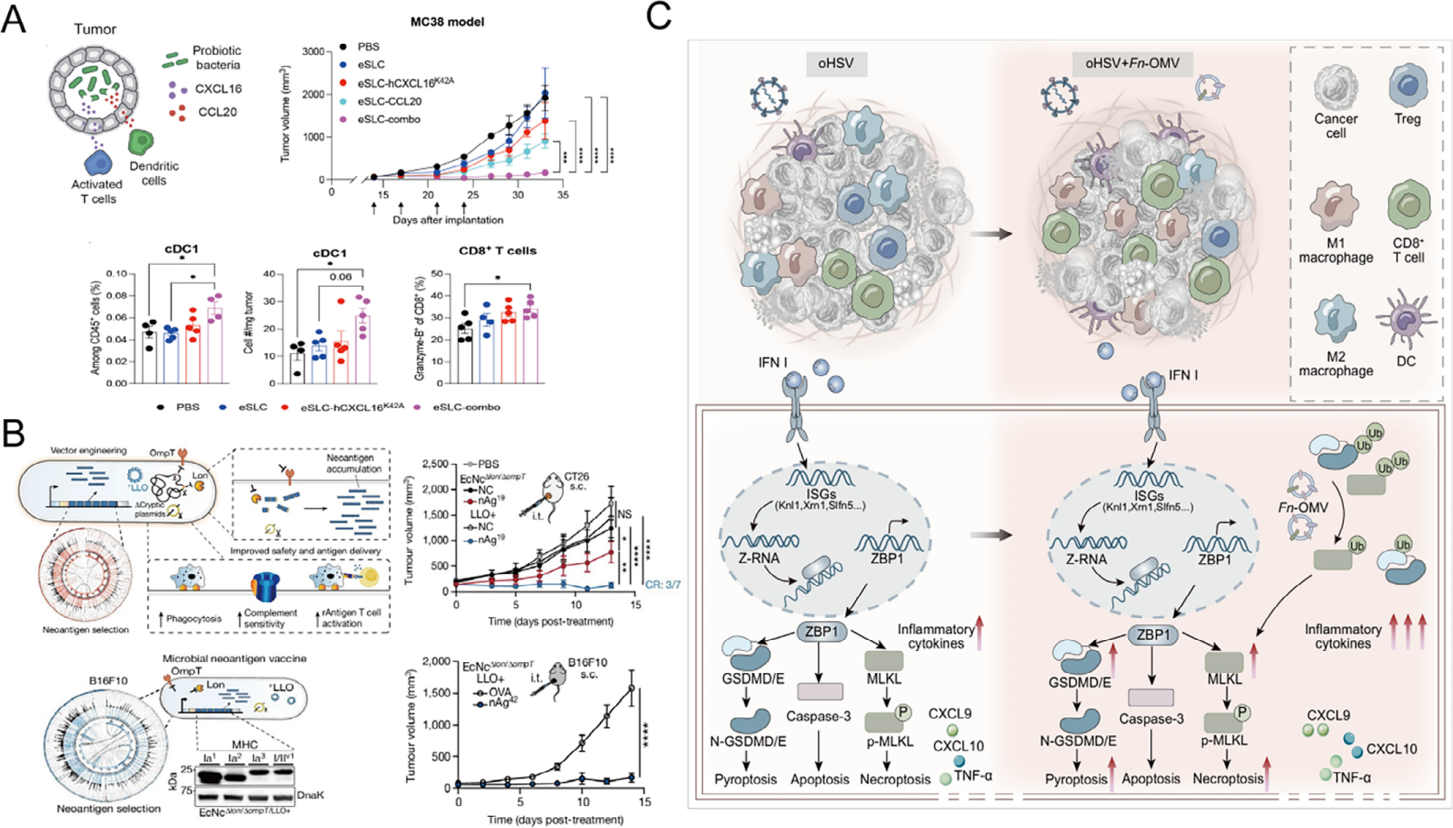
A) Schematic illustration of the eSLC‐combo strategy. ECNs encoding CXCL16 and CCL20 effectively recruit and activate dendritic cells and T cells within the tumor microenvironment. This approach significantly enhances immune cell infiltration and activation, resulting in superior tumor regression compared to direct administration of recombinant CXCL16/CCL20 protein alone. Reproduced with permission.^[^ ^50^ ^]^ Copyright 2023, American Association for the Advancement of Science. B) Schematic diagram of EcNc^Δ^ *^lon^* ^/Δ^ *^ompT^* ^/LLO+^nAg^19^, designed to express tumor‐specific neoantigens along with listeriolysin O. This design stimulates robust CD4⁺ and CD8⁺ T‐cell responses, achieving significant tumor growth inhibition and preventing tumor rechallenge in B16F10 melanoma and CT26 colorectal cancer mouse models. Reproduced with permission.^[^ ^51^ ^]^ Copyright 2024, Springer Nature. C) Schematic representation of Fn‐OMVs enhancing oHSV‐induced PANoptosis. The addition of Fn‐OMVs upregulates expression of GSDMD, GSDME, and MLKL proteins by inhibiting their degradation pathways, thereby amplifying tumor cell death and increasing proinflammatory cytokine release.^[^ ^56^ ^]^ Copyright 2024, Springer Nature.

Engineered bacteria provide multiple advantages in cancer immunotherapy, including direct tumor cell killing and stimulation of both innate and adaptive immune responses. They also serve as versatile carriers for delivering therapeutic agents to the TME.^[^ ^12^, ^108^ ^]^ *Escherichia coli* Nissle 1917 (ECN) is an ideal platform for therapeutic engineering due to its non‐pathogenic nature, selective tumor colonization, and ease of genetic modification.^[^ ^109^ ^]^ Several engineered ECN strains have been developed to enhance antitumor immune responses, targeting both innate and adaptive immunity to transform immunosuppressive TMEs into immune‐active states. For instance, ECN engineered with a synchronized lysis circuit (SLC) produces and releases a nanobody antagonist of CD47 (CD47nb) within the TME.^[^ ^12^ ^]^ The SLC triggers controlled bacterial lysis via a bacteriophage lysis protein, releasing CD47nb, which blocks the “don't eat me” signal on cancer cells, enabling macrophage‐mediated phagocytosis. This approach significantly increases T‐cell infiltration in both treated and untreated tumors. In a subsequent study, SLC‐encoded ECN were engineered to produce chemokines CXCL16 and CCL20 (eSLC‐combo), enhancing tumor regression in mouse models by recruiting and activating DCs and T cells.^[^ ^50^ ^]^ This strategy outperformed direct administration of recombinant CXCL16 protein, demonstrating a more potent antitumor response (**Figure** **4**A).

Another prominent ECN‐derived strain is SYNB1891, which is engineered to secrete cyclic di‐AMP (CDA), a potent agonist of the Stimulator of Interferon Genes (STING) pathway, bridging innate and adaptive immunity.^[^ ^52^ ^]^ STING activation triggers type I interferon (IFN‐I) signaling, which enhances tumor antigen presentation, promotes T‐cell activation, and boosts overall antitumor immunity. In a B‐cell lymphoma mouse model, intratumoral administration of SYNB1891 led to complete tumor rejection, demonstrating its potential for clinical translation.

In another approach, ECN was engineered to produce L‐arginine, an amino acid critical for T‐cell activation and proliferation.^[^ ^53^ ^]^ Elevated L‐arginine levels within the TME promote tumor‐infiltrating lymphocyte (TIL) expansion, reduce immunosuppressive regulatory T cells (Tregs) and myeloid‐derived suppressor cells (MDSCs), and enhance responses to anti‐PD‐L1 checkpoint inhibitors. In preclinical models, 74% of mice treated with L‐arginine‐producing ECN combined with checkpoint blockade achieved complete tumor regression, highlighting the strategy's efficacy in overcoming immunosuppressive barriers.

ECN has also been modified to express tumor‐specific neoantigens and listeriolysin O (LLO) in a strain designated EcNc^Δlon/ΔompT/LLO+^ nAg^19^.^[^ ^51^ ^]^ This modification triggers robust CD4^+^ and CD8^+^ T‐cell responses by providing continuous neoantigen exposure, which enhances antigen presentation and fosters long‐term immune memory. In melanoma and colorectal cancer mouse models, this strain significantly reduced tumor burden and prevented tumor rechallenge, indicating its potential to induce durable antitumor immunity (Figure 4B). The inclusion of LLO, a pore‐forming toxin, facilitates antigen release and immune cell activation, further amplifying the therapeutic effect.

#### 2.3.2 Outer Membrane Vesicles

The replicative capacity of live bacteria poses risks of unintended infections, making outer membrane vesicles (OMVs) a safer alternative with high immunogenicity and improved biosafety.^[^ ^11^, ^110^, ^111^ ^]^ OMVs, derived from bacterial membranes, are rich in PAMPs that activate innate immunity and DCs.^[^ ^111^ ^]^ For example, double‐layered membrane vesicles (DMVs) from *Salmonella Typhimurium* VNP20009 were used to enhance photothermal therapy (PTT) by coating mesoporous polydopamine (MPD) nanoparticles, forming a core‐shell structure (MPD@DMV).^[^ ^54^ ^]^ This structure combines the photothermal properties of MPD with the immunogenic effects of OMVs. DMVs act as immune adjuvants, with bacterial membrane components (e.g., proteins, LPS, and other microbe‐associated molecular patterns) stimulating antitumor immune responses. MPD@DMV nanoparticles accumulate in tumor tissues, where laser irradiation triggers the MPD core to generate heat for tumor ablation, while DMVs amplify the immune response, resulting in enhanced antitumor effects. In another study, OMVs from engineered photosynthetic bacteria (*Rhodopseudomonas palustris*), modified with maleimide (MAL) to capture antigens, passively target tumor‐draining lymph nodes (TDLNs) due to their small size.^[^ ^55^ ^]^ This accumulation enhances antigen presentation to DCs, promoting T‐cell activation and a robust antitumor immune response.

OMVs also augment oncolytic virus‐based therapies, such as oncolytic herpes simplex virus‐1 (oHSV) therapy.^[^ ^56^ ^]^ OMVs derived from *Fusobacterium nucleatum* (Fn‐OMVs) enhance oHSV‐mediated PANoptosis by upregulating execution proteins like Gasdermin D (GSDMD), Gasdermin E (GSDME), and mixed lineage kinase domain‐like (MLKL) protein through inhibition of their degradation (Figure 4C). The combination of Fn‐OMVs and oHSV converts M2 tumor‐associated macrophages to the M1 phenotype, reduces Treg populations, and enhances the efficacy of PD‐1/PD‐L1 checkpoint blockade therapy.

### 2.4 PAMP‐Mimetic Molecules

Inspired by the ability of bacteria and viruses to activate pattern recognition receptors (PRRs), synthetic PRR agonists have significantly advanced cancer immunotherapy. These agonists, including toll‐like receptor (TLR)^[^ ^112^ ^]^ and cGAS‐STING agonists,^[^ ^113^ ^]^ mimic pathogen‐ or damage‐associated molecular patterns (PAMPs or DAMPs) to stimulate robust immune responses.^[^ ^114^ ^]^ They promote IFN‐I production,^[^ ^115^ ^]^ recruit immune cells, or activate antigen‐presenting cells (APCs) by targeting various PRR pathways.^[^ ^114^ ^]^ Intratumoral administration of these agonists directly reverses the immunosuppressive tumor microenvironment (TME) and enhances antigen presentation, offering a targeted approach to bolster antitumor immunity.^[^ ^116^ ^]^

#### 2.4.1 STING Agonist

The cGAS‐STING pathway is activated by natural or synthetic cyclic dinucleotides (CDNs), which trigger immune responses critical for tumor therapy.^[^ ^117^ ^]^ For example, 5,6‐dimethylxanthenone‐4‐acetic acid (DMXAA) not only enhances antigen presentation but also exerts direct tumor‐killing effects in mouse models.^[^ ^57^ ^]^ Intratumoral DMXAA injection promotes tumor regression, improves survival, reduces tumor vessel size, and increases tumor‐specific T‐cell populations in vivo. Another synthetic STING agonist, ADU‐S100, induces potent immune activation, but its efficacy is often limited by STING promoter hypermethylation in certain tumors, particularly melanoma.^[^ ^118^ ^]^ This epigenetic silencing hinders sustained cGAS‐STING pathway activation, reducing long‐term tumor suppression. Combining ADU‐S100 with 5‐aza‐2′‐deoxycytidine (5AZADC), a DNA methyltransferase inhibitor, reverses hypermethylation, restoring STING expression.^[^ ^58^ ^]^ This combination significantly increases MHC class I expression and IFN‐β release, enhancing antigen presentation and T‐cell‐mediated responses, thus promoting durable antitumor effects in cancers with epigenetic STING silencing.

**Figure 5:**
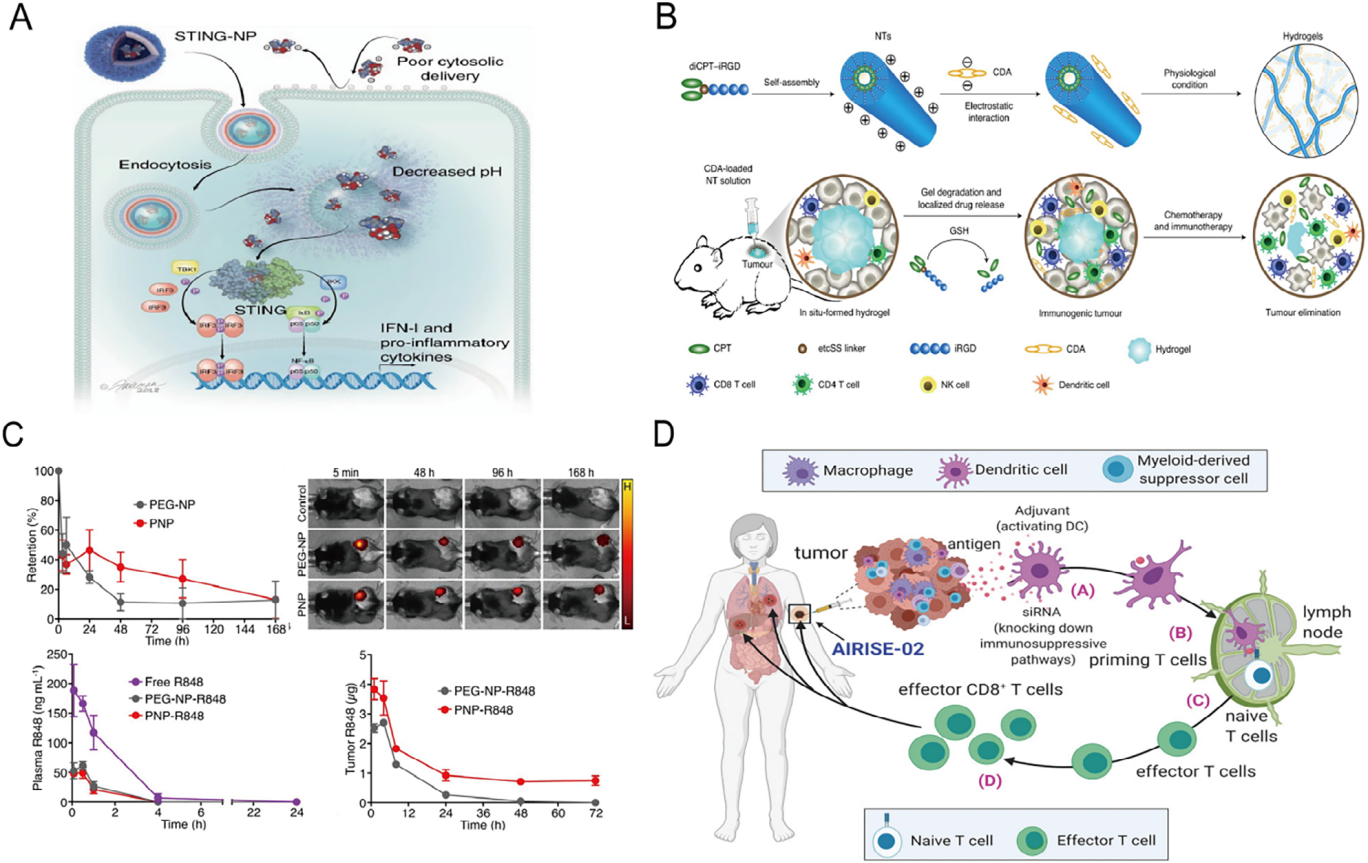
A) Schematic illustration of STING‐NPs facilitating enhanced intracellular delivery of 2′3'‐cGAMP. Following cellular uptake, the acidic environment within endosomes triggers the release of cGAMP, activating the STING pathway and subsequently inducing the production of type I interferons. Reproduced with permission.^[^ ^59^ ^]^ Copyright 2019, Springer Nature. B) Schematic illustration depicting the formation of CDA‐NTs via CPT–iRGD conjugation. These hydrogels sustain the delivery of CPT and CDNs. CPT‐induced DNA damage increases cytosolic DNA accumulation, thereby activating the cGAS‐STING pathway, while CDNs directly stimulate STING signaling. Reproduced with permission.^[^ ^60^ ^]^ Copyright 2020, Springer Nature. C) Schematic of polymeric nanoparticles encapsulating R848 (PNP‐R848), demonstrating enhanced tumor‐cell interaction and prolonged retention within the tumor microenvironment. Reproduced with permission.^[^ ^64^ ^]^ Copyright 2021, Springer Nature. D) Schematic illustration of AIRISE‐02 nanoparticles co‐delivering siSTAT3 and CpG for cancer vaccination. CpG enhances dendritic cell activation and antigen presentation, while siSTAT3 suppresses immunosuppressive pathways. This combined approach significantly augments T‐cell‐mediated antitumor immunity.^[^ ^65^ ^]^ Copyright 2021, Wiley‐VCH.

Despite their immune‐activating potential, STING agonists face challenges with cellular uptake. To improve intracellular delivery and protect agonists from degradation, endosomolytic polymersomes encapsulating 2′3'‐cGAMP (STING‐NPs) were developed (**Figure** **5**A).^[^ ^59^ ^]^ In a melanoma model, intratumoral STING‐NPs significantly increased Ifnb1 gene expression (encoding IFN‐β) and elicited a stronger IFN response compared to free cGAMP, leading to improved tumor regression and survival with minimal systemic toxicity. Similarly, lipidoid nanoparticles (LNPs) encapsulating cGAMP enable endosomal escape and cytoplasmic STING activation.^[^ ^16^ ^]^ Pretreatment with doxorubicin (DOX) induces tumor cell death, releasing antigens captured by LNPs for MHC class I presentation, activating CD8^+^ T cells. This approach enhances antigen cross‐presentation and TME modulation, resulting in robust, long‐lasting T‐cell responses.

To further enhance STING agonist efficacy, increasing DNA damage is an effective complementary strategy. Camptothecin (CPT), a DNA‐damaging agent, promotes cytosolic DNA accumulation, which cGAS senses to produce CDNs, activating the STING pathway (Figure 5B).^[^ ^60^ ^]^ CDNs loaded into nanotubes formed from a camptothecin–iRGD conjugate (diCPT–iRGD), termed CDN‐loaded nanotubes (CDA‐NTs), create a hydrogel for sustained release under physiological conditions. Fluorescence‐labeled CDA‐NTs remain detectable for up to 35 days post‐injection, ensuring long‐term drug retention at the tumor site, which leads to prolonged survival, durable immune responses, and significant tumor regression.

Mn^2^⁺ enhances cGAS‐STING activation by increasing cGAS sensitivity to DNA and improving cGAMP‐STING binding affinity.^[^ ^119^ ^]^ However, high Mn^2^⁺ concentrations can cause cytotoxicity.^[^ ^120^ ^]^ Mn^2^⁺‐based nanoparticles, such as MnO_2_^[^ ^21^ ^]^ and manganese phosphate nanoparticles,^[^ ^121^ ^]^ enable controlled delivery to the TME. PEGylated manganese phosphate nanoclusters increase IFN‐β and IL‐6 release and promote dendritic cell maturation in vitro and in vivo.^[^ ^121^ ^]^ However, Mn^2^⁺ nanoparticles alone often yield limited outcomes due to insufficient cytosolic DNA damage^[^ ^122^ ^]^ and reactive oxygen species (ROS) generation.^[^ ^123^ ^]^ To address this, SN38, a DNA‐damaging chemotherapeutic, was encapsulated in diselenide bond‐bridged mesoporous silica nanoparticles coated with Mn^2^⁺ and epigallocatechin gallate (EGCG).^[^ ^61^ ^]^ This combination synergistically activates the cGAS‐STING pathway, while selenium (Se) and EGCG scavenge ROS, reducing oxidative stress and protecting dendritic cells from Mn^2^⁺‐induced cytotoxicity, thereby enhancing immune activation and therapeutic efficacy.

#### 2.4.2 TLR Agonist

Polyinosinic:polycytidylic acid (poly(I:C)), a synthetic double‐stranded RNA, activates TLR3, located in the endosomal compartments of dendritic cells and macrophages, which primarily recognize viral double‐stranded RNA.^[^ ^62^ ^]^ Intratumoral administration of poly(I:C) increases macrophage and CD8^+^ T‐cell infiltration into tumor tissue while reducing immunosuppressive regulatory T cells (Tregs). However, monotherapy with poly(I:C) or other TLR agonists often yields limited efficacy due to insufficient systemic immune activation. To enhance therapeutic outcomes, researchers have explored multi‐pathway immune activation. Combining poly(I:C) with R848, a TLR7/8 agonist, promotes consistent production of T‐cell‐attracting chemokines and polarization of macrophages to the proinflammatory M1 phenotype.^[^ ^124^ ^]^ In mouse models, polymeric nanocapsules loaded with both poly(I:C) and R848 demonstrated superior antitumor efficacy and reduced lung metastasis compared to either agent alone, highlighting the benefit of synergistic TLR activation.

TLR4, expressed on the surface of immune cells, recognizes bacterial lipopolysaccharides (LPS).^[^ ^125^ ^]^ Glucopyranosyl lipid A (GLA), a synthetic analogue of LPS, binds TLR4 with reduced toxicity compared to LPS.^[^ ^126^ ^]^ In clinical trials, intratumoral injection of G100, a GLA‐containing formulation, induces proinflammatory changes in the TME, leading to tumor regression with a favourable safety profile.^[^ ^63^ ^]^ This approach provides immune‐mediated tumor inhibition while minimizing systemic toxicity. Combining G100 with adoptive cell therapy (ACT) or a lentiviral vector (Zvex) has achieved complete regression of both primary and secondary tumors in preclinical models, demonstrating its potential to enhance systemic antitumor immunity.^[^ ^127^ ^]^

R848 (resiquimod) and CpG oligodeoxynucleotides (CpG ODNs) are widely used TLR agonists that activate TLR7/8 and TLR9, respectively, primarily on dendritic cells, which are critical for antigen presentation in immunotherapy.^[^ ^128^ ^]^ Systemic administration of free agonists often results in dissemination, reducing their concentration at the tumor site and limiting efficacy. To address this, platelet membrane‐cloaked nanoparticles (PNP‐R848) have been developed to enhance R848 delivery by improving tumor cell interaction and prolonging drug retention within the TME (Figure 5C).^[^ ^64^ ^]^ These nanoparticles promote dendritic cell maturation and increase CD4^+^ and CD8^+^ T‐cell infiltration, enhancing local immune activation.

Despite their potential, TLR agonists often produce localized immune responses that are insufficient for controlling distant metastatic tumors.^[^ ^129^ ^]^ Immunosuppressive cells, such as myeloid‐derived suppressor cells (MDSCs), which secrete IL‐10 and TGF‐β, can further dampen CpG‐induced responses.^[^ ^130^ ^]^ To overcome these limitations, combining CpG with STAT3 siRNA mitigates immunosuppression and promotes immunogenic cell death.^[^ ^65^, ^131^ ^]^ In a bilateral tumor model, AIRISE‐02 nanoparticles delivering both siSTAT3 and CpG (siSTAT3–CpG–NP) delayed tumor growth. STAT3 inhibition enhanced CpG's adjuvanticity, leading to increased CD8^+^ T‐cell proliferation and an improved CD8^+^/Treg ratio.^[^ ^65^ ^]^ Furthermore, combining CpG nanoparticles with immune checkpoint inhibitors achieved curative effects and prevented tumor recurrence in a melanoma model, demonstrating the potential for systemic immunity (Figure 5D).

### 2.5 Chemotherapeutic Drugs and Nano‐Inducers

Chemotherapy remains a cornerstone of cancer treatment, utilizing agents such as doxorubicin (DOX),^[^ ^132^ ^]^ oxaliplatin,^[^ ^133^ ^]^ and gemcitabine^[^ ^29^ ^]^ to induce DNA damage and apoptosis in cells. However, these drugs non‐selectively target both malignant and healthy cells, leading to significant side effects, including organ toxicity and immunosuppression.^[^ ^134^ ^]^ Even localized injections can result in systemic circulation, exacerbating these adverse effects.^[^ ^135^ ^]^ Additionally, the short half‐lives of chemotherapeutic agents limit their sustained therapeutic impact.^[^ ^136^ ^]^ To address these challenges, advanced delivery systems, such as polymer‐encapsulated nanoparticles,^[^ ^137^ ^]^ pH‐responsive nanodisks,^[^ ^138^ ^]^ and hydrogel^[^ ^139^ ^]^ have been developed to enable targeted drug release within the TME, enhancing efficacy while minimizing toxicity.

#### 2.5.1 Chemotherapeutic Drugs

A notable example involves the co‐delivery of DOX and the PD‐L1‐binding peptide DPPA‐1 via an injectable, thermo‐responsive hydrogel.^[^ ^66^ ^]^ This hydrogel facilitates sustained DOX release at the tumor site, where DOX induces immunogenic cell death. Concurrently, DPPA‐1 blocks the PD‐L1 immune checkpoint, enhancing CD8^+^ T‐cell infiltration into the TME, thereby amplifying antitumor immunity.

**Figure 6:**
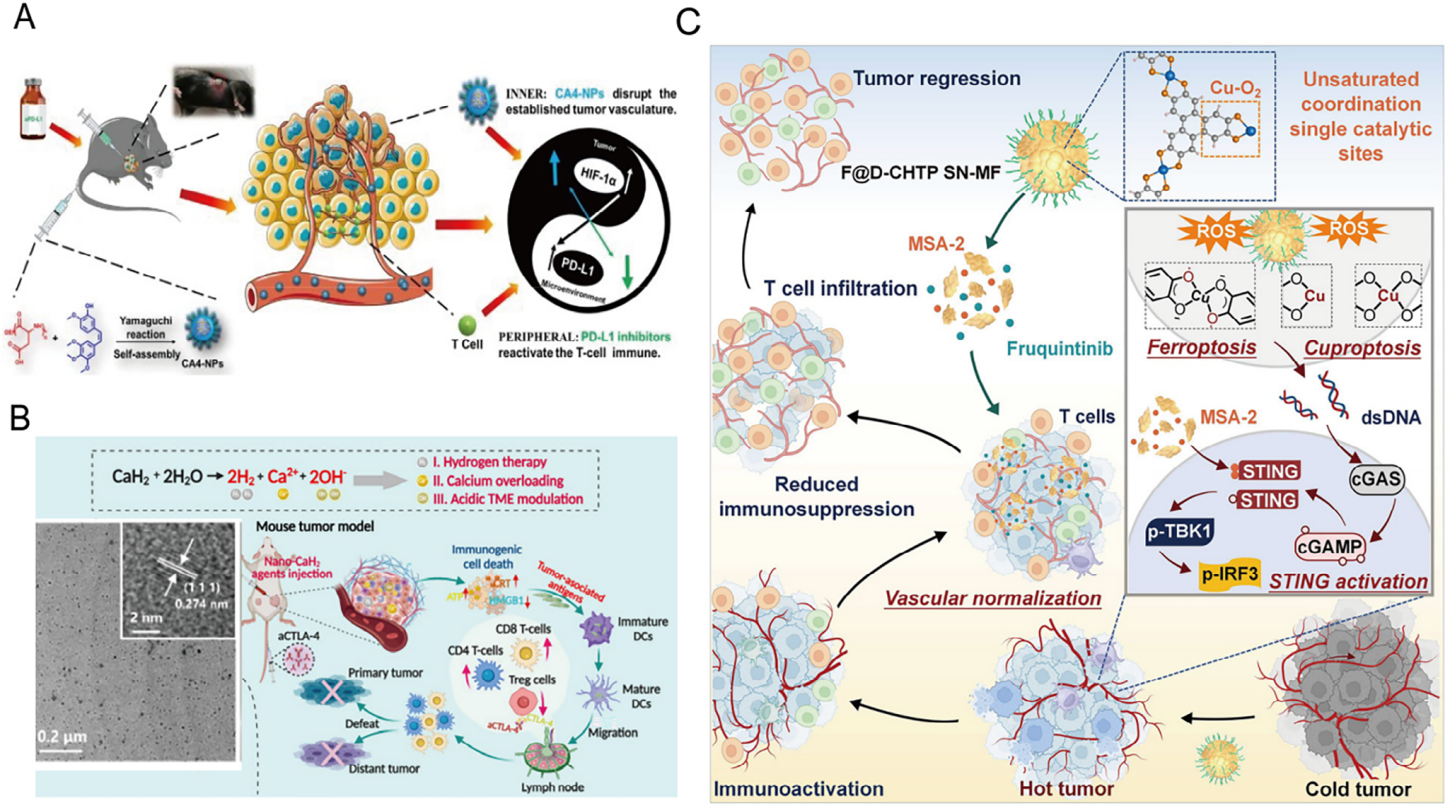
A) Schematic illustration of CA4‐NPs designed to enhance tumor retention of the vascular‐disrupting agent CA4P. CA4P treatment induces tumor hypoxia, leading to upregulated expression of HIF‐1α and PD‐L1. Combination therapy with intratumoral anti‐PD‐L1 counters this hypoxia‐driven immunosuppression, thereby enhancing T‐cell‐mediated antitumor immune responses.^[^ ^67^ ^]^ Copyright 2020, American Chemical Society. B) Schematic and representative TEM image illustrating nano‐CaH_2_ and its mechanism of action in hydrogen‐immunotherapy within a mouse xenograft model. Nano‐CaH_2_ reacts with water to release hydrogen gas (H_2_), calcium ions (Ca^2^⁺), and hydroxide ions (OH−), neutralizing tumor acidity and triggering immunogenic cell death (ICD). This process induces oxidative stress, mitochondrial dysfunction, and immune activation, significantly promoting activation of DCs as well as infiltration of CD8⁺ and CD4⁺ T cells. Reproduced with permission.^[^ ^20^ ^]^ Copyright 2022, Cell Press. C) Schematic representation illustrating the multifunctional nanozyme F@D‐CHTP SN‐MF, featuring exposed Cu‐O_2_ sites, in combination with fruquintinib (a VEGFR inhibitor) and MSA‐2 (a STING agonist). This nanozyme induces ferroptosis and cuproptosis through reactive oxygen species (ROS) generation. Concurrently, MSA‐2 activates the STING signaling pathway, and fruquintinib normalizes tumor vasculature, enhancing immune cell infiltration. This synergistic approach effectively remodels the tumor microenvironment from an immunosuppressive “cold” state to an immunologically active “hot” state, promoting robust T‐cell infiltration and potent antitumor efficacy.^[^ ^68^ ^]^ Copyright 2025, Cell Press.

Unlike traditional chemotherapeutic agents that directly inhibit cancer cell proliferation through DNA damage, Combretastatin A‐4‐phosphate (CA4P) targets tumor vasculature, disrupting nutrient and oxygen supply.^[^ ^140^ ^]^ This vascular disruption also upregulates CXCL12, enhancing the recruitment of conventional dendritic cells (cDCs). To improve CA4P's efficacy and reduce systemic side effects, CA4P‐loaded nanoparticles (CA4‐NPs) were developed to enhance tumor retention (**Figure** **6**A).^[^ ^67^ ^]^ The hypoxic conditions induced by CA4P increase hypoxia‐inducible factor 1‐alpha (HIF‐1α) expression, which upregulates PD‐L1. Combining CA4‐NPs with intratumoral anti‐PD‐L1 antibodies significantly prolongs survival and promotes tumor regression by counteracting this immunosuppressive mechanism.

#### 2.5.2 Nano‐Inducers

Nanoparticles can function as direct therapeutic agents in cancer treatment. Metal‐based nanoparticles, such as gold nanoparticles (AuNPs)^[^ ^141^ ^]^ and iron oxide nanoparticles,^[^ ^142^ ^]^ induce cancer cell death by generating reactive oxygen species (ROS), leading to oxidative stress and apoptosis.^[^ ^143^ ^]^ Calcium‐based nanoparticles, such as calcium carbonate, cause calcium overload, disrupting mitochondrial function and triggering apoptosis. For example, calcium hydride (nano‐CaH_2_) nanoparticles react with water to produce hydrogen gas (H_2_), calcium ions (Ca^2^⁺), and hydroxide ions (OH−), neutralizing the acidic TME (Figure 6B).^[^ ^20^ ^]^ This H_2_‐mediated oxidative stress induces mitochondrial dysfunction, ICD, apoptosis, and tumor cell calcification. In a CT26 bilateral tumor model, nano‐CaH_2_ outperformed results in a 4T1 model, and its combination with anti‐CTLA4 therapy significantly enhanced antitumor immune responses in the 4T1 tumor model.

Another study developed a multifunctional single‐site nanozyme (F@D‐CHTP SN‐MF) with exposed Cu‐O_2_ sites (Figure 6C).^[^ ^68^ ^]^ This nanozyme coordinates ferroptosis, cuproptosis, STING pathway activation, and tumor vasculature normalization. By generating abundant ROS, it induces ICD and enhances tumor immunogenicity. Co‐delivery of MSA‐2 (a STING agonist) and fruquintinib (a VEGFR inhibitor) synergistically activates innate immunity and remodels the TME, improving immune cell infiltration and antitumor responses.

Initially, ICD was primarily associated with apoptosis.^[^ ^144^ ^]^ However, recent research highlights alternative cell death pathways, such as pyroptosis,^[^ ^145^ ^]^ ferroptosis (iron‐dependent),^[^ ^146^ ^]^ and cuproptosis (copper‐dependent),^[^ ^147^ ^]^ which also trigger robust ICD, releasing tumor antigens and DAMPs to enhance antitumor immunity.^[^ ^148^ ^]^ For instance, sodium chloride nanoparticles (SCNPs) induce pyroptosis by activating caspase‐1, increasing IL‐1β secretion, and causing lysosomal cathepsin B release, K⁺ efflux, and osmotic imbalance, leading to cell lysis.^[^ ^69^ ^]^ This pyroptotic ICD promotes DC maturation, antigen cross‐presentation, and robust T‐cell‐mediated immunity in C3H/HeN mice. Another study induced pyroptosis via mitochondrial calcium overload through the cytochrome C‐caspase‐3/GSDME pathway, demonstrating diverse molecular triggers for immunogenic outcomes.^[^ ^70^ ^]^

### 2.6 Physical Therapy

Physical therapies, including radiotherapy,^[^ ^75^, ^149^ ^]^ photodynamic therapy,^[^ ^150^ ^]^ and sonodynamic therapy^[^ ^151^ ^]^ are integral to clinical oncology due to their ability to directly ablate tumor cells through localized treatment. However, their efficacy is limited by the need to restrict doses to prevent damage to adjacent healthy tissues.^[^ ^11^, ^152^ ^]^ Additionally, these therapies often fail to induce robust immune responses due to low lymphocyte infiltration and the immunosuppressive TME.^[^ ^153^ ^]^ Combining these therapies with immune modulators or adjuvants is critical to mitigate immunosuppression and enhance systemic immune responses.

#### 2.6.1 Phototherapy

**Figure 7:**
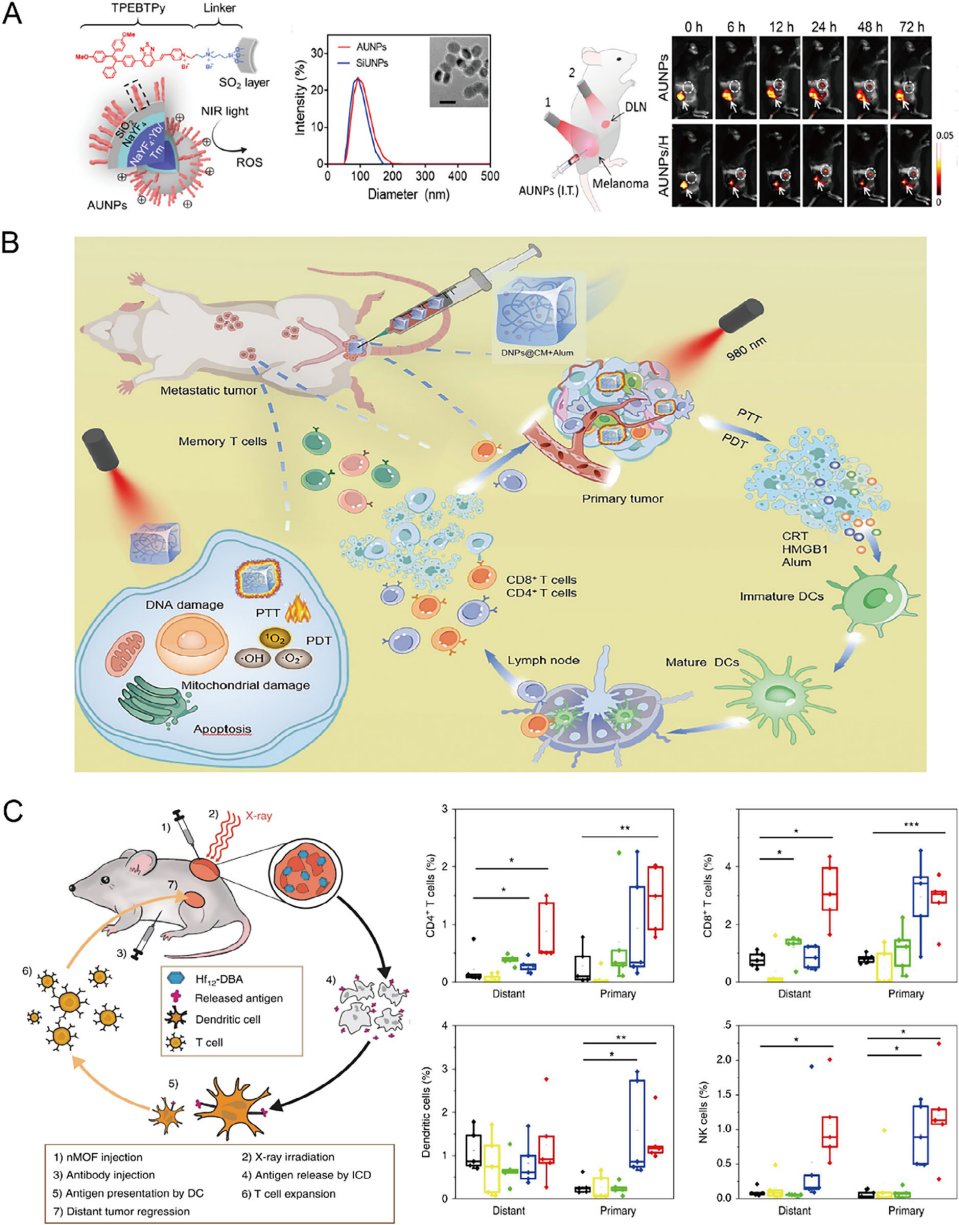
A) Schematic illustration of the design and characterization of AUNP. Fluorescence imaging of draining lymph nodes demonstrates significantly increased fluorescent signals in mice treated with AUNPs under irradiation compared to AUNP treatment alone. This indicates enhanced capture and uptake of tumor‐associated antigens by antigen‐presenting cells. Reproduced with permission.^[^ ^72^ ^]^ Copyright 2020, American Association for the Advancement of Science. B) Schematic illustration of the therapeutic mechanism of DNPs@CM+Alum hydrogel optimized for dual‐mode phototherapy. Intratumoral injection of DNPs@CM+Alum facilitates strong intramolecular charge transfer, resulting in broad NIR absorption and suppressed fluorescence, thereby enhancing ROS generation upon NIR irradiation. The incorporation of aluminum adjuvant gel further amplifies phototherapy‐induced immune activation. Copyright 2018, Springer Nature. Reproduced with permission.^[^ ^73^ ^]^ Copyright 2024, Wiley‐VCH. C) Schematic depiction of Hf_12_‐DBA nanoplate‐mediated immune activation combined with anti‐PD‐L1 therapy. Intratumoral administration of Hf_12_‐DBA nanoplates followed by X‐ray irradiation induces immunogenic cell death, which, in synergy with anti‐PD‐L1 therapy, promotes extensive infiltration and activation of DCs, T cells, and NK cells within the TME. Copyright 2018, Springer Nature. Reproduced with permission.^[^ ^75^ ^]^ Copyright 2018, Springer Nature.

Photodynamic therapy (PDT) and photothermal therapy (PTT) are advanced, light‐based treatment modalities that leverage photosensitizing or thermosensitive agents to target tumors with high precision.^[^ ^101^, ^154^ ^]^ PDT relies on the activation of photosensitizers by light to produce ROS, which trigger tumor cell apoptosis or necrosis.^[^ ^154^, ^155^ ^]^ Despite its advantages, PDT is hindered by limited light penetration, restricting its efficacy to superficial tumors, and insufficient immune activation to sustain robust antitumor responses.^[^ ^156^ ^]^ To address these limitations, AIEgen‐coupled upconversion nanoparticles (AUNPs) have been developed for dual‐mode ROS generation and enhanced tissue penetration (**Figure** **7**A).^[^ ^72^ ^]^ Under high‐dose light irradiation, AUNPs induce immunogenic cell death (ICD), releasing tumor‐associated antigens that stimulate immune responses. Low‐power irradiation enhances T‐cell activation, significantly reducing immunosuppressive cells, such as regulatory T cells (Tregs), and promoting overall immune activation. This dual‐mode approach leverages the unique properties of AUNPs to overcome PDT's depth limitations and enhance its immunotherapeutic potential.

In PTT, thermosensitive agents activated by NIR light increase local temperatures to induce tumor cell death.^[^ ^157^ ^]^ To minimize damage to surrounding healthy tissues, one study employed a mild temperature of ≈45 °C for PTT.^[^ ^71^ ^]^ However, mild PTT can upregulate immunosuppressive proteins, such as programmed death‐ligand 1 (PD‐L1), which dampen antitumor immunity. To counteract this, a thermo‐responsive lipid hydrogel was developed to co‐deliver the photothermal agent IR820 and an anti‐PD‐L1 antibody. This hydrogel ensures controlled release upon mild laser irradiation, prolonging agent retention at the tumor site. By inducing tumor cell death and blocking PD‐L1, this strategy converts immunologically “cold” tumors into “hot” ones, enhancing responsiveness to ICB therapy. This approach demonstrates the potential of combining PTT with immunotherapy to overcome immunosuppressive barriers in the tumor microenvironment.

The individual limitations of PDT and PTT, such as restricted light penetration and suboptimal immune activation, have prompted the development of combined PDT/PTT strategies to achieve synergistic effects while minimizing damage to healthy tissues.^[^ ^158^ ^]^ An acceptor–donor–acceptor (A–D–A)‐structured nanoaggregate, DNPs@CM, was developed for dual phototherapy, leveraging strong intramolecular charge transfer (ICT) for broad NIR absorption and suppressed fluorescence to enable deeper tissue penetration (Figure 7B).^[^ ^73^ ^]^ When combined with an aluminum adjuvant gel in vivo, DNPs@CM sustains tumor ICD and effectively suppresses tumor metastasis, offering a robust platform for integrated phototherapy.

#### 2.6.2 Radiotherapy

Radiotherapy employs ionizing radiation to treat cancer by inducing DNA damage and cell death in tumor cells.^[^ ^159^ ^]^ Unlike photodynamic therapy (PDT), which uses light‐activated photosensitisers,^[^ ^160^ ^]^ radiotherapy relies on high‐energy X‐rays or other radiation sources to target tumors. However, its efficacy is limited by the need to minimize damage to surrounding healthy tissues and its often‐limited ability to stimulate robust immune responses. To address these challenges, nanomaterials engineered as radiosensitizers and innovative radiation delivery strategies have been developed to enhance therapeutic outcomes and synergize with immunotherapies.

Certain nanomaterials are designed as radiosensitizers to amplify the effects of radiotherapy. Hafnium‐based nanoscale metal‐organic frameworks (MOFs), specifically Hf‐DBA, outperform clinically investigated hafnium oxide (HfO_2_) nanoparticles due to their highly porous structures and thin nanoplate morphologies.^[^ ^75^ ^]^ The high atomic number of hafnium enhances X‐ray absorption, generating photoelectrons and Auger electrons that increase reactive oxygen species (ROS) production under irradiation. The porous structure of Hf‐DBA MOFs facilitates efficient ROS diffusion, further contributing to their radiosensitizing capabilities. This heightened ROS production amplifies DNA damage in cancer cells, leading to more effective tumor cell killing. Additionally, Hf‐DBA induces immunogenic cell death (ICD), which activates dendritic cells (DCs) and promotes tumor‐specific T‐cell responses, enhancing the efficacy of immune checkpoint blockade therapies, anti‐PD‐L1 (Figure 7C).

Recent studies suggest that heterogeneous radiotherapy (RT), which delivers varying radiation doses across different tumor regions, significantly enhances immune activation compared to traditional homogeneous RT.^[^ ^74^ ^]^ High‐dose RT regions induce robust ICD, releasing tumor antigens that stimulate immune responses. However, high doses can impair the ability of APCs, such as dendritic cells, to cross‐present antigens in tumor‐draining lymph nodes (TDLNs). In contrast, low‐to‐moderate dose regions preserve APCs function and promote T‐cell infiltration and activation. This spatial variation in dosing optimizes the balance between direct tumor cell killing and immune stimulation, fostering effective antitumor T‐cell expansion. By reprogramming the immunosuppressive tumor microenvironment (TME), heterogeneous RT enhances synergy with immune checkpoint inhibitors, leading to stronger and more durable antitumor responses.

## 3 Clinical Translation

### 3.1 FDA‐Approved In Situ Vaccines

#### 3.1.1 Bacillus Calmette–Guérin

**Figure 8:**
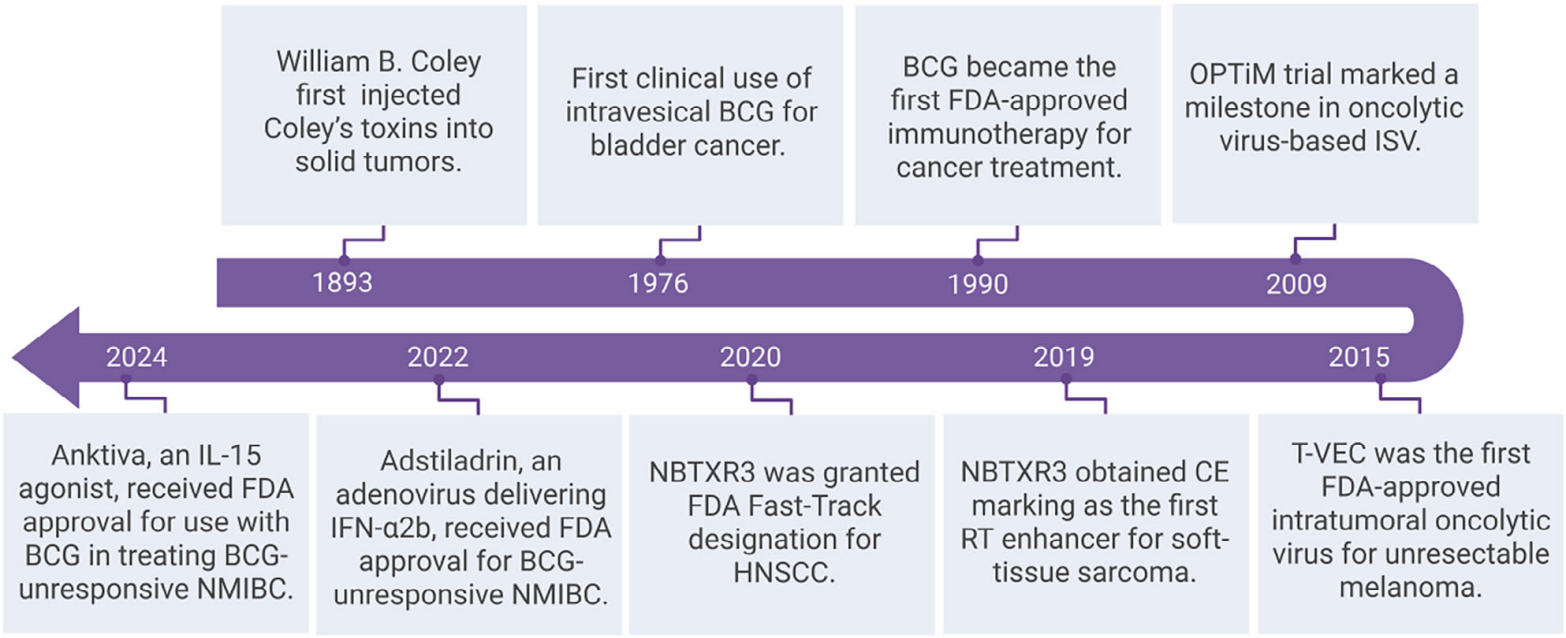
Historical Development of Clinically Approved In Situ Vaccines. ISV: In situ vaccination; BCG: Bacillus Calmette‐Guérin; FDA: U.S. Food and Drug Administration; T‐VEC: Talimogene laherparepvec, CE: Conformité Européenne; RT: Radiotherapy; HNSCC: Head and Neck Squamous Cell Carcinoma; NMIBC: Non‐muscle‐invasive bladder cancer; IFN‐α2b: Interferon alpha‐2b; IL‐15: Interleukin‐15. Created with BioRender.com.

Bacillus Calmette–Guérin is a live attenuated strain of *Mycobacterium bovis*. It was originally developed as a tuberculosis vaccine in the early 20th century. Its potential in cancer therapy emerged in the mid‐20th century when BCG‐treated mice showed increased resistance to tumor implantation.^[^ ^161^ ^]^ In 1976, intravesical BCG administration marked a milestone in cancer immunotherapy by demonstrating efficacy against bladder cancer (**Figure** **8**).^[^ ^162^ ^]^ A pivotal clinical trial conducted in the late‐1980s, compared intravesical BCG to doxorubicin in patients with superficial bladder cancer and revealed that BCG significantly reduced tumor recurrence rates, leading to its approval by the U.S. FDA in 1990 as the first cancer immunotherapy.^[^ ^163^ ^]^

Despite the efficacy of tumor resection followed by BCG therapy, up to 50% of patients experience tumor recurrence,^[^ ^164^ ^]^ highlighting the need for improved strategies. Combination therapies have shown promise in addressing this limitation. For instance, combining BCG with chemotheraputic agents such as mitomycin C (NCT01442519)^[^ ^165^ ^]^ or gemcitabine (NCT04179162)^[^ ^166^ ^]^ demonstrated higher remission rates and longer remission durations compared to BCG alone. On the other hand, BCG‐unresponsive non‐muscle‐invasive bladder cancer (NMIBC) could be treated with other immunotherapy strategies such as immune checkpoint inhibitors. Anti‐PD‐1 monotherapy, Pembrolizumab, has shown promising antitumor activity in BCG‐unresponsive NMIBC (NCT02625961). Multi‐drug regimens, including atezolizumab combined with cisplatin/gemcitabine and BCG, have been investigated to enhance pathological complete remission rates and prolong patient survival (NCT04630730).

Finally, to improve patient tolerability and reduce local side effects of BCG therapy, hyaluronic acid was co‐administered to patients showing reduced side effects (NCT02207608).

#### 3.1.2 Talimogene Laherparepvec

Talimogene laherparepvec (T‐VEC), the first FDA‐approved oncolytic virus‐based immunotherapy, is derived from herpes simplex virus type 1 (HSV‐1). Engineered with deletions of ICP34.5 and ICP47 genes to limit replication in normal cells, T‐VEC selectively infects and lyses cancer cells. It also incorporates the granulocyte‐macrophage colony‐stimulating factor (GM‐CSF) gene to enhance dendritic cell recruitment and antigen presentation.^[^ ^167^ ^]^ Phase I clinical trials in advanced melanoma demonstrated a favorable safety profile,^[^ ^168^ ^]^ while subsequent Phase II trials confirmed T‐VEC's oncolytic activity and systemic efficacy, achieving a 26% response rate with durable effects in both injected and uninjected lesions.^[^ ^169^ ^]^ The pivotal Phase III OPTiM trial (NCT00769704), launched in 2009, demonstrated significantly higher durable response rates and complete response rates with T‐VEC compared to GM‐CSF alone, with no treatment‐related mortality.^[^ ^170^ ^]^ These results led to FDA approval in October 2015 for melanoma with cutaneous and lymph node involvement.

Further studies have explored T‐VEC in combination with immune checkpoint inhibitors, including nivolumab (NCT04330430), ipilimumab (NCT01740297), pembrolizumab (NCT02509507, NCT02965716), atezolizumab (NCT03256344), and panitumumab (NCT04163952). These combinations enhanced antitumor responses without significant additional toxicity.

On the other hand, a Phase I/II clinical trial (NCT03555032) combining T‐VEC and melphalan, administered through isolated limb perfusion in stage IIIb/c and IVa/b melanoma, is currently ongoing. Additionally, a Phase II trial combining T‐VEC with paclitaxel in triple‐negative breast cancer (TNBC) patients undergoing surgery (NCT02779855) demonstrated enhanced immune responses, favourable pathological outcomes, and a manageable safety profile, supporting further investigation into T‐VEC's role in combination therapies for TNBC.^[^ ^171^ ^]^

Radiotherapy combined with T‐VEC was found to be well‐tolerated and may augment immune responses (NCT02453191). Ongoing trials are evaluating this combination in soft tissue sarcoma (NCT02923778, NCT06660810).

#### 3.1.3 Other FDA‐Approved ISV

In the past five years, three additional in situ vaccines have received FDA approval: NBTXR3, ADSTILADRIN, and Anktiva. NBTXR3, a hafnium oxide nanoparticle‐based radio‐enhancer, received European CE mark approval in 2019 for soft tissue carcinoma and FDA approval in 2020 for head and neck squamous cell carcinoma (HNSCC). Its ability to enhance radiotherapy makes it particularly valuable for elderly patients (≥65 years) with locally advanced, platinum‐ineligible HNSCC, who often cannot tolerate cisplatin‐based chemoradiotherapy. A Phase III trial (NCT04892173) is evaluating a combination of NBTXR3 and cetuximab with radiotherapy elderly population. Additionally, when combined with anti‐PD‐1 therapy, NBTXR3 showed potential in overcoming resistance to immune checkpoint inhibitors and improving therapeutic responses in HNSCC (NCT03589339).

ADSTILADRIN (nadofaragene firadenovec), approved in 2022 for BCG‐unresponsive NMIBC, is a non‐replicating adenoviral vector encoding human interferon alfa‐2b cDNA. An ongoing Phase II trial (NCT06545955) is assessing its efficacy in combination with chemotherapy (gemcitabine and docetaxel) or immunotherapy (pembrolizumab) for high‐grade BCG‐unresponsive NMIBC.

Anktiva (N‐803) is an interleukin‐15 receptor agonist approved in 2024 for BCG‐unresponsive NMIBC, is also being explored for other cancers. A Phase II trial (NCT04247282) evaluating Anktiva combination with neoantigen delivering adenovirus (TriAd5 vaccine) and bispecific Bintrafusp alfa (anti TGF‐β and anti‐PD‐L1) as neoadjuvant therapy for HPV‐unrelated HNSCC reported encouraging recurrence‐free survival outcomes.^[^ ^172^ ^]^ Ongoing Phase II trials are investigating Anktiva in combination therapies for glioblastoma (NCT06061809) and colorectal cancer (NCT04491955) using subcutaneous administration, underscoring its versatility in cancer immunotherapy.

### 3.2 Ongoing In Situ Vaccines in Clinical Trials

Many types of ISVs have shown promising outcomes in both preclinical and clinical studies (**Table** **2**), demonstrating their potential in boosting anti‐tumor immunity and reducing the risk of metastasis. For example, except for T‐VEC, other oncolytic viruses are being widely explored in clinical trials, particularly in combination with immune checkpoint inhibitors. V937 (Coxsackievirus A21) combined with pembrolizumab (anti‐PD‐1) in advanced melanoma achieved a 47% objective response rate (ORR) and 22% complete response rate (CR) (NCT02565992). Similarly, oncolytic Coxsackievirus A21 was evaluated in combination with ipilimumab (anti‐CTLA‐4), achieving a 30% ORR, with higher efficacy observed in checkpoint inhibitor‐naïve patients (NCT02307149).

**Table 2:** Summary of In Situ Vaccines Evaluated in Clinical Trials.

Clinical Trial Number	Functionalized Agents	Cancer Types
NCT00880867	Poly‐ICLC Plus Low Dose Local Radiation	Low Grade Recurrent B and T Cell Lymphoma
NCT01397708	Adenovirus Vector Engineered to Express hIL‐12	Melanoma
NCT02225366	LL37 Peptide	Melanoma
NCT06430515	cisplatin, oxaliplatin	Advanced solid cancers (lung and liver cancers)
NCT05838729	RiMO‐301+hypofractionated radiation+PD‐L1	Unresectable, recurrent, or metastatic head‐neck cancer
NCT03788083	TriMix	Early Breast Cancer
NCT04612504	SynOV1.1 + Atezolizumab	Hepatocellular carcinoma (HCC)
NCT06014086	PH‐762	Cutaneous Carcinoma
NCT00668512	alpha‐Gal glycosphingolipids	Advanced Melanoma
NCT03435952	Clostridium Novyi‐NT + Pembrolizumab	Advanced solid tumor
NCT04781725	INT230‐6 (cisplatin, vinblastine, shao)	Breast Cancer
NCT05076760	MEM‐288 Oncolytic Virus (CD40L and type I interferon) + Nivolumab	Non‐Small Cell Lung Cancer (NSCLC)
NCT04260360	NanoDoce (small (submicron) particles of docetaxel)	Renal Cell Carcinoma
NCT04270864	Tilsotolimod (a TLR‐9 Agonist),Ipilimumab + Nivolumab	Advanced Cancers
NCT06048367	CNSI‐Fe(II)	Advanced solid tumors

Recently, bacteria‐based therapies have also gained attention as innovative oncolytic strategies. Salmonella typhimurium strain SGN1, engineered to overexpress L‐methioninase, has demonstrated tumor‐specific colonization, metastasis inhibition, and tumor regression in clinical trials (NCT05103345; NCT05038150). Meanwhile, engineered E.coli minicells delivering the pore‐forming protein perfringolysin O have been tested for advanced solid tumors via intratumoral injection (NCT05901285). Additionally, Clostridium novyi‐NT, a toxin‐deficient anaerobic bacterial strain capable of selectively colonizing hypoxic tumor regions, was evaluated in combination with pembrolizumab (anti‐PD‐1) (NCT03435952). A single injection of C. novyi‐NT combined with pembrolizumab demonstrated a manageable toxicity profile and encouraged anticancer activity in patients with solid tumors.

On the other hand, immune‐stimulating agonists such as TLR agonists, STING agonists, and RNA‐based immune stimulants are evaluated to activate innate and adaptive immune responses. For instance, tilsotolimod (IMO‐2125), a TLR9 agonist, was evaluated as an intratumoral injection in combination with ipilimumab (anti‐CTLA‐4) in PD‐1 inhibitor‐refractory advanced melanoma (NCT02644967). A Phase III clinical trial (ILLUMINATE‐301, NCT03445533) evaluated the efficacy of combining tilsotolimod with ipilimumab versus ipilimumab alone in patients with refractory melanoma. The addition of tilsotolimod did not result in an improvement in overall survival. MK‐2118, a non‐cyclic dinucleotide STING agonist, was evaluated via intratumoral and subcutaneous administration with or without pembrolizumab (NCT03249792). BO‐112, a nanoplexed poly(I:C) RNA agonist, was evaluated in a phase I trial (NCT02828098) in patients with PD‐1‐refractory tumors. Intratumoral BO‐112 combined with nivolumab or pembrolizumab led to partial responses in melanoma and renal cell carcinoma patients, with increased CD8^+^ T cell infiltration and type I IFN activation.

Moreover, Biomolecules such as cytokines, peptides, and chemokines are widely explored for tumor microenvironment modulation. In a Phase I study (NCT00977145), intratumoral IFN‐γ increased T cell‐recruiting chemokines but failed to enhance T cell infiltration, highlighting the limitations of single‐agent therapy in overcoming immune exclusion. In a Phase II trial, L19‐IL2/L19‐TNF demonstrated high local tumor control and systemic immune activation, making it a promising neoadjuvant strategy for inoperable melanoma. Additionally, LTX‐315, an oncolytic peptide (NCT01986426), induced tumor regression and abscopal effects by enhancing tumor antigen release and CD8^+^ T cell infiltration, positioning it as a potential checkpoint inhibitor combination therapy.

## 4 Conclusion and Prospective

ISVs offer a transformative approach to cancer immunotherapy by harnessing the tumor as an endogenous antigen source to elicit robust systemic immune responses. ISVs can enhance immune activation while minimizing systemic toxicity through intratumoral administration of immune modulators such as cytokines, agonists, oncolytic viruses, and engineered bacteria.

The FDA‐approval of T‐Vec after it has shown promising antitumor efficacy for melanoma highlights ISVs’ potential in cancer therapy. Nevertheless, significant hurdles, including tumor heterogeneity, delivery constraints, safety risks, and variable patient responses, must be overcome to fully realize the clinical promise of ISVs.

Tumor heterogeneity, encompassing both intra‐tumoral variations within a single tumor and inter‐tumoral differences across patients, presents a formidable challenge to the efficacy of ISV. Diverse antigen expression profiles and the immunosuppressive nature of the TME can impede uniform immune activation, particularly in tumors with low immunogenicity, such as pancreatic cancer. This variability hinders the ability of ISVs to elicit a consistent and robust immune response, as poorly immunogenic tumors often resist immune cell infiltration and activation.

Optimizing delivery remains a critical hurdle in ISV development, primarily due to insufficient penetration and retention of therapeutic agents in larger or heterogeneous tumors, which compromises their effectiveness. For mRNA‐based formulations, key challenges include the inherent instability of mRNA, which is highly susceptible to degradation by nucleases in the TME, significantly shortening its functional half‐life. Even when mRNA is successfully internalized by cells, its translation into functional immune‐stimulating factors such as cytokines or costimulatory molecules can be impaired by the immunosuppressive TME, resulting in suboptimal immune activation. Additionally, advanced delivery systems like lipid nanoparticles (LNPs) struggle to precisely target tumor‐resident APCs or tumor cells, often leading to off‐target delivery or inadequate uptake. Hydrogels, employed for sustained release, face difficulties in maintaining consistent release kinetics, risking rapid burst release or unintended diffusion from the injection site, which may provoke local inflammation or toxicity. These multifaceted challenges underscore the complexity of achieving robust and consistent therapeutic outcomes in ISVs.

The use of engineered bacteria in ISVs raises significant safety concerns that demand meticulous management, particularly the risks of systemic infection and horizontal gene transfer. These risks are evident in studies involving Clostridium bacteria, where the potential for bacteria to spread beyond the tumor or transfer engineered genes to other microbes has been noted. For ISVs, which typically rely on intratumoral injection, higher local doses may be feasible compared to systemic administration; however, rigorous monitoring is essential to detect and manage potential systemic dissemination and associated side effects. Genetic kill switches, developed through synthetic biology, provide an additional layer of control by regulating bacterial growth and preventing uncontrolled proliferation. Particular caution is required when administering bacterial‐based therapies to immunocompromised patients, as their diminished immune capacity increases the likelihood of adverse reactions.

Emerging technologies hold significant promise for transforming ISV development. AI‐driven antigen profiling can enhance the identification of immunogenic tumor antigens, facilitating the creation of personalized ISV formulations. For instance, machine learning algorithms analysing genomic and transcriptomic data have demonstrated the ability to predict patient responses, as evidenced in studies of immune checkpoint inhibitors. Similarly, implantable microdevices offer precise delivery of multiple therapeutics and real‐time monitoring of tumor responses, potentially improving ISV efficacy and control.

To advance ISVs, future research should prioritize the integration of cutting‐edge technologies to address current limitations. Developing robust biomarkers for patient stratification, leveraging AI and genomic profiling, will enable treatments tailored to individual tumor profiles, enhancing therapeutic precision. Improving delivery systems to enhance tumor penetration and retention is critical to boosting ISV efficacy. Real‐time monitoring tools, such as circulating tumor DNA (ctDNA) analysis and advanced imaging, will facilitate adaptive treatment strategies by providing insights into ongoing immune responses. By addressing these challenges and harnessing innovative technologies, ISVs have the potential to become a cornerstone of personalized cancer immunotherapy.

## Conflict Of Interest

The authors declare no conflict of interest.
